# Ginsenoside Rg3 nanoparticles with permeation enhancing based chitosan derivatives were encapsulated with doxorubicin by thermosensitive hydrogel and anti-cancer evaluation of peritumoral hydrogel injection combined with PD-L1 antibody

**DOI:** 10.1186/s40824-022-00329-8

**Published:** 2022-12-09

**Authors:** Hao Wu, Guoli Wei, Lixia Luo, Lingchang Li, Yibo Gao, Xiaobin Tan, Sen Wang, Haoxiao Chang, Yuxi Liu, Yingjie Wei, Jie Song, Zhenhai Zhang, Jiege Huo

**Affiliations:** 1 Affiliated Hospital of Integrated Traditional Chinese and Western Medicine, Nanjing University of Chinese Medicine, 210023 Nanjing, China; 2 Jiangsu Province Academy of Traditional Chinese Medicine, 210028 Nanjing, China; 3 School of Material Science and Chemical Engineering, Chuzhou University, 239000 Chuzhou, China; 4 Department of Oncology, Nanjing Lishui District Hospital of Traditional Chinese Medicine, Nanjing, China; 5 Department of Neurology, Beijing Tiantan Hospital, Capital Medical University, Beijing, China

**Keywords:** Chitosan, Thermo-sensitive hydrogel, Immunochemotherapy, Immunogenic cell death, Doxorubicin, Ginsenoside Rg3

## Abstract

**Background:**

Combination of chemotherapy and immune checkpoint inhibitor therapy has greatly improved the anticancer effect on multiple malignancies. However, the efficiency on triple-negative breast cancer (TNBC) is limited, since most patients bear “cold” tumors with low tumor immunogenicity. Doxorubicin (DOX), one of the most effective chemotherapy agents, can induce immunogenic cell death (ICD) and thus initiating immune response.

**Methods:**

In this study, to maximize the ICD effect induced by DOX, chitosan and cell-penetrating peptide (R6F3)-modified nanoparticles (PNPs) loaded with ginsenoside Rg3 (Rg3) were fabricated using the self-assembly technique, followed by co-encapsulation with DOX based on thermo-sensitive hydrogel. Orthotopic tumor model and contralateral tumor model were established to observe the antitumor efficacy of the thermo-sensitive hydrogel combined with anti-PD-L1 immunotherapy, besides, the biocompatibility was also evaluated by histopathological.

**Results:**

Rg3-PNPs strengthened the immunogenic cell death (ICD) effect induced by DOX. Moreover, the hydrogel co-loading Rg3-PNPs and DOX provoked stronger immune response in originally nonimmunogenic 4T1 tumors than DOX monotherapy. Following combination with PD-L1 blocking, substantial antitumor effect was achieved due to the recruitment of memory T cells and the decline of adaptive PD-L1 enrichment.

**Conclusion:**

The hydrogel encapsulating DOX and highly permeable Rg3-PNPs provided an efficient strategy for remodeling immunosuppressive tumor microenvironment and converting immune “cold” 4T1 into “hot” tumors.

**Supplementary Information:**

The online version contains supplementary material available at 10.1186/s40824-022-00329-8.

## Introduction

Immunotherapy is a promising anticancer treatment with huge potential. Checkpoint inhibitor therapy (ICT) against programmed death ligand-1 (PD-L1) and programmed cell death-1 (PD-1), has exhibited a prominent response in various malignancies. However, just a small fraction of triple-negative breast cancer (TNBC) patients could respond to ICT, mainly attributable to immunologically “cold” tumors and immunosuppressive tumor microenvironment (TME) [1, 2]. “Cold tumors” are characterized by the absence of T-cell infiltration, low MHC I expression, low mutational load, and deficient PD-L1 expression [3]. Therefore, additional therapies to remold TME remain a thorny challenge for improving the antitumor efficacy. Modulation of T cells to create an inflamed “hot tumor” for maximum effective PD-1/L1 therapy is desirable.

Immunogenic cell death (ICD) is a kind of cell death which induced the antitumor immune response. ICD is well recognized to reverse the low immunogenicity of “cold” tumors by unleashing damage-associated molecular patterns (DAMPs) [4], including the surface exposure of calreticulin (CRT), the secretion of adenosine triphosphate (ATP) and high mobility group box 1 protein (HMGB1), followed by the maturation of the antigen-presenting dendritic cells (DCs) and initiating a cascade process leading to antigen specific T-cell infiltration [5]. The binding of HMGB1 to TLR4 stimulates inflammatory responses [6]. Such vaccine-like effect in situ would provoke adaptive immune responses, facilitating the transition of “cold tumors” into “hot tumors” [7], and finally reshaping the immunosuppressive microenvironment and eradicating cancer cells.

Nowadays, researchers found that some tumor treatment strategies could induce ICD, which makes the tumor visible for the immune system, including hyperthermia, radiation, photodynamic therapy, and chemotherapy, such as the anthracycline doxorubicin (DOX) [8]. ICD effect caused by chemotherapy plays a vital role in improving immunotherapy. DOX has been widely used in chemotherapy for various malignancies, not only inducing cancer cell death but also stimulating ICD-induced immunity [9]. However, the immunogenicity of DOX to eliminate tumor cell is weak, yielding inadequate DAMPs to initiate satisfied anti-cancer immune response by itself [10]. And tumor relapse can often be observed in clinic.

A number of research indicated the traditional Chinese medicine component exhibited the great potential in the adjuvant treatment of cancer. Ginsenoside Rg3 is a ginseng saponin that has antitumor and immune-modulatory activity. In addition, the combination of Rg3 with chemotherapeutic drugs like paclitaxel, docetaxel and DOX has gained attention for outstanding antitumor activities in various types of malignant tumors [11]. Recent research indicated that Rg3 could enhance the ICD effect induced by DOX and ignited the immunity system in acute myeloid leukemia (AML) mice [12]. However, insolubility and lack of targeting greatly limited the penetration of Rg3 in tumors, thus affecting the combination outcome of DOX and Rg3.

To solve this problem, nanoparticles delivery system was introduced in our study. Chitosan (CS) is a naturally occurring and abundantly available polysaccharide with excellent biocompatibility and biodegradability [13], and has received much attention in the biomedical and pharmaceutical fields. In addition, the structure of CS with free -NH_2_ and -OH is amenable to chemical modifications that can improve the properties for application [14]. Plenty of studies utilized this characteristic to improve the treatment including active targeting, lysosomal escape, and TME response and also to promote drug penetration [15–17]. Recently, many research confirmed that the lower drug penetration is the key issue for immunotherapeutic treatment failure including TNBC [18, 19]. Therefore, chemotherapy with deep tumor penetration is desired. Cell-penetrating peptides (CPPs) have attracted extensive attention with the ability to carry nanoparticles into the cell, thereby improving the curative effectiveness [20]. Here, CPPs with six arginine and three phenylalanine (R_6_F_3_) were used to modify the CS to encapsulate Rg3 and to increase the penetration of Rg3. Besides, the amphiphilic property of R_6_F_3_ also contributed to the stability of nanoparticles, thus avoiding the premature release of Rg3.

**Scheme 1:**
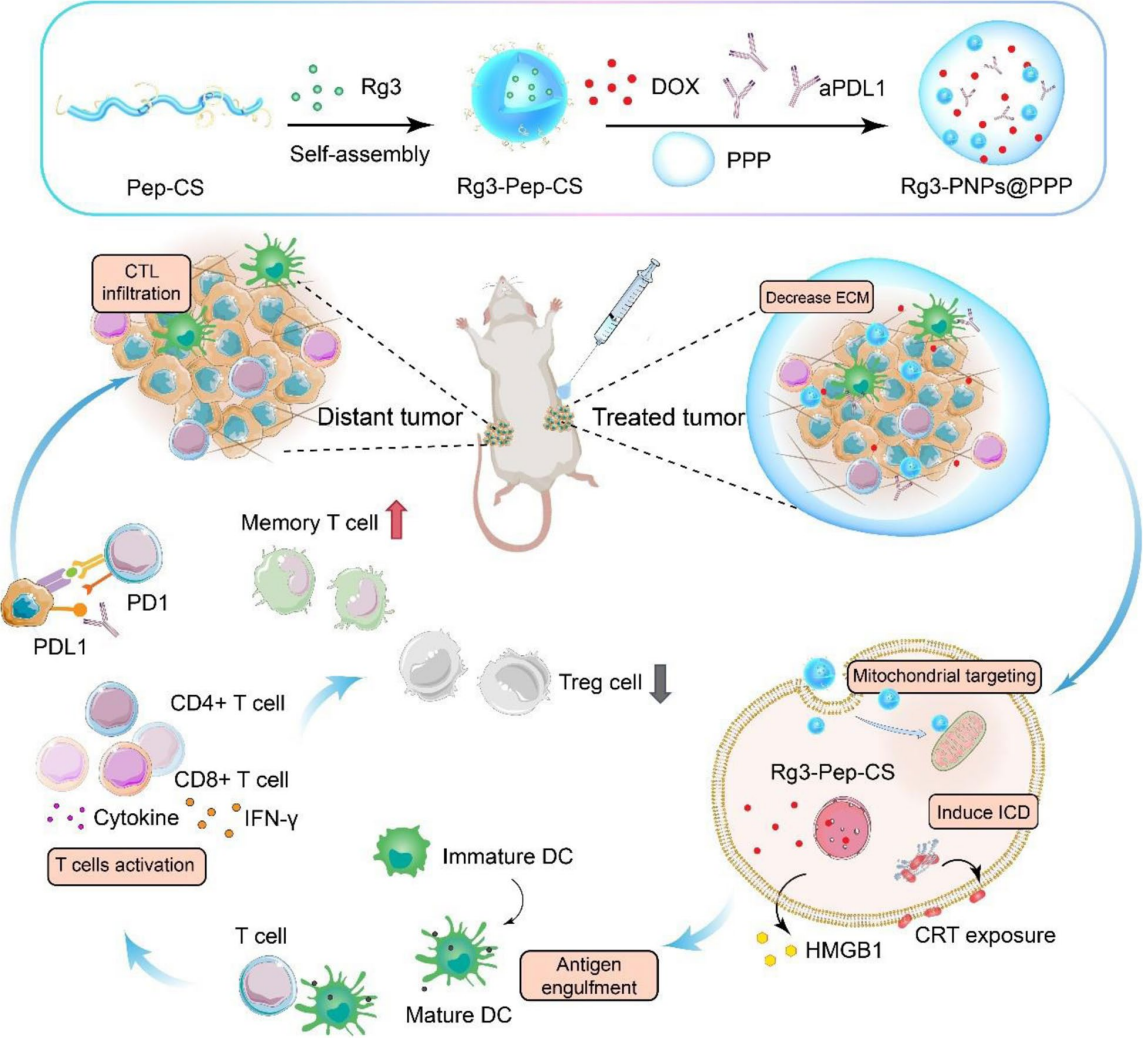
Schematic illustration of the thermo-sensitive hydrogel formation and the inhibition of local chemotherapy combined with immunotherapy on abscopal tumors

To minimize the systemic toxicity and short circulation of DOX, Rg3-PNPs and anti-PD-L1, while leveraging the collaborative therapeutic effect, we presented the localized chemo-immunotherapy based on thermo-sensitive hydrogels to boost the anti-tumor immunotherapeutic efficacy for 4T1 tumor (Scheme 1), which lacks estrogen receptor, progesterone receptor and human epidermal growth factor receptor2, regarded as a typically TNBC model [21]. The peritumorally injected thermo-sensitive hydrogels were loaded with DOX, Rg3-PNPs and anti-PD-L1. Delivery of Rg3 by the transmembrane peptide and chitosan could decrease solid stress and degrade extracellular matrix of tumor. Besides, since R_6_F_3_ was located on the surface of the nanoparticles, Rg3 could penetrate into the tumor cells to the greatest extent, followed by mitochondrial targeting due to the positive charge of the nanoparticle, thereby reinforcing the ICD effect induced by DOX. Therefore, abundant tumor cell debris was exposed, with subsequent DC maturation and T cell activation. Local chemotherapy combined with PD-L1 blockade further intensified the potency to eradicate abscopal tumors. In addition, the local combination therapy also decreased the tumor volume and significantly prolonged survival time in the orthotopic 4T1 model. All in all, our drug delivery system offers a facile and reliable approach to decrease the immunosuppression and to improve the checkpoint-blocking therapy for 4T1 tumors.

## Materials and methods

### Cells and reagents

4T1 cells (ATCC) were maintained in 10% v/v FBS supplied RPMI-1640 medium (Keygen Biotech, Nanjing, China), with 100 mg/mL of streptomycin and 100 units/mL penicillin at 37°C humidified environment with 5% CO_2_ supply. R_6_F_3_ oligopeptide was synthesized by Nanjing peptide Biotechnology Co., Ltd (Nanjing, China). PLGA-PEG-PLGA (MW = 1400-1500-1400) was obtained from Jinan Daigang Biomaterial Co., Ltd (Jinan, China). Doxorubicin hydrochloride (DOX·HCl) was purchased from Zhejiang Hisun pharmaceutical Co., Ltd (Taizhou, China). Chitosan was purchased from Zhejiang Golden shell pharmaceutical Co., Ltd (Taizhou, China). Ginsenoside Rg3 (Rg3) were purchased from Tianjin Wanxianghengyuan Technology Co., Ltd (Tianjin, China). (R)-(+)-α-lipoic acid (LA), 1-ethyl-3(3-di-methylaminopropyl) carbodiimide hydrochloride (EDCI) and 1-hydroxybenzotriazole monohydrate (HOBT) were purchased from Aladdin Reagent Co., Ltd (Shanghai, China). Coumarin-6 (CUR) and 4’,6-diamidino-2-phenylindole (DAPI) were obtained from Shanghai Aladdin Bio-Chem Technology Co., Ltd (Shanghai, China). Chitosan (CS) was purchased from Nantong green god biological engineering Co., Ltd (Nantong, China). α-SMA, CCL2, Ki-67, CRT and HMGB1 antibodies were all obtained from Abcam (Shanghai, China). Anti-PD-L1 (αPD-L1) was purchased from Bioxcell (West lebanon, NH, USA).

### Preparation of co-delivery drug system

#### Peptides-modified Chitosan (R_6_F_3_-CS)

R_6_F_3_-CS was prepared according to the previous literature with a slight modification [22]. Briefly, R_6_F_3_ (139.7 mg, 0.1 mmoL) were dissolved in phosphate buffer solution (pH 7.4), and then incubated with EDCI and HOBT at the room temperature for 4 h. CS (0.1 mmoL) was also dissolved in PBS and stirred for 48 h. The mixture was isolated by distilled water for 24 h in 3500 Da MWCO dialysis bag and then freeze-dried to yield the product, denoting as R_6_F_3_-CS. The obtained product was characterized by ^1^ H-NMR and FT-IR. Nitrogen Content was measured by elemental analyzer. Therefore, the grafting rate (GA) of R_6_F_3_ can be calculated according to the Eq. (1).

$$Nitrogen\;Content\;(\%)=\frac{\mathrm{AM}(\mathrm N)\times\mathrm{AA}({\mathrm N}_{\mathrm{PEP}})\times\mathrm{GA}(\%)+\mathrm{AM}(\mathrm N)\times\mathrm{AA}({\mathrm N}_{\mathrm{CS}})}{\mathrm{MW}(\mathrm{PEP})\times\mathrm{GA}(\%)+\mathrm{MW}(\mathrm{CS})}$$

1 $$Nitrogen\;Content\;(\%)=\frac{14\times27\times\mathrm{GA}(\%)+14\times1}{1395\times GA(\%)+161}$$

Where AM(N) is the atomic mass of nitrogen; AA(N_PEP_) is the atom amount of nitrogen in the peptides; GA represents the grafting rate of the peptides, and AA(N_CS_) is the atom amount of nitrogen in the glucosamine unit of CS. MW(PEP) is the molecule weight of PEP; MW(CS) is molecule weight of the glucosamine unit in CS.

#### Preparation of Rg3-loaded nanoparticle

Rg3-loaded nanoparticle was prepared by combining sonication and dialysis method [23]. In brief, 20 mg of CS or R_6_F_3_-CS powder was dissolved in 20 mL deionized water, and then mixed with 4 mg of Rg3 dissolved in methanol with stirring at room temperature overnight. Following this, the solution was sonicated by probe-type ultrasonicator at 150 W for 3 min, the pulse was work for 3 s with 6 s interval. Afterwards, the mixture was dialyzed against distilled water using a dialysis bag (3500 Da) for 24 h to remove the free drug and methanol, finally yielding Rg3-loaded nanoparticle. Nanoparticle made up of Rg3 and CS was denoted as Rg3-NPs, while nanoparticle made up of Rg3 and R_6_F_3_-CS was named as Rg3-PNPs.

#### Preparation of hydrogel delivery system

The thermo-sensitive hydrogel (PLGA-PEG-PLGA, PPP) was applied for co-encapsulation of Rg3-PNPs and DOX. PPP was dissolved in 0.9% NaCl solution. Free Rg3, DOX, DOX + Rg3 or Rg3-PNPs + DOX were weighed and stirred with PPP in 0.9% NaCl solution at 4 °C until polymer was dissolved to form a clear solution [24]. Followed with continuous stir, the Rg3@PPP, DOX@PPP, Rg3 + DOX@PPP and Rg3-PNPs + DOX@PPP were obtained respectively. The stability of blank PPP and Rg3-PNPs + DOX@PPP was further evaluated using dynamic light scattering (DLS).

### Evaluation of particle size, zeta potential and morphology

The particle size of Rg3-NPs and Rg3-PNPs were measured by dynamic laser-light scattering instrument (Brookhaven Instruments, USA) at room temperature. The morphology was observed by transmission electron microscopy (TEM) using a HT 7700 (Hitachi, Japan) after negative staining with phosphor tungstic acid solution.

### Drug-loading and encapsulation efficiency

The Rg3 concentration was determined using high-performance-liquid chromatography (HPLC, Agilent 1260, Agilent Technologies, Santa Clara, CA, USA) [25]. The amount of Rg3 loaded was determined by dissolving nanoparticles in acetonitrile. The solution was sonicated for 30 min to damage the structure and then filtered through a 0.45 μm membrane filter. The drug loading (DL) and entrapment efficiency (EE) were calculated using the following Eqs. (2 and 3):

2 $$DL\;(\%)\;=\frac{\mathrm{Weight}\;\mathrm{of}\;\mathrm{Rg}3\;\mathrm{in}\;\mathrm{nanoparticles}}{\mathrm{Weight}\;\mathrm{of}\;\;\mathrm{nanoparticles}}\times100\%$$

3 $$EE\;(\%)\;=\frac{\mathrm{Weight}\;\mathrm{of}\;\mathrm{Rg}3\;\mathrm{in}\;\mathrm{nanoparticles}}{\mathrm{Weight}\;\mathrm{of}\;\;\mathrm{the}\;\mathrm{feeding}\;\mathrm{Rg}3}\times100\%$$

### In vitro release of DOX and Rg3 from PPP

The in vitro release behaviors of Rg3-PNPs + DOX and Rg3-PNPs + DOX@PPP were also investigated. Briefly, the Rg3-PNPs + DOX@PPP were slowly added into the tube and incubated at 37 °C to convert the hydrogels, meanwhile, Rg3-PNPs + DOX were introduced into a dialysis bag. Afterwards, 30 mL PBS was added into each formulation as a release medium. The samples were kept in the air bath incubator at 37 °C with the shake rate of 100 rpm [12]. The PBS was acquired and analyzed at 1, 2, 4, 8, 12, 24, 48 and 72 h. The release profiles of DOX and Rg3 were analyzed by HPLC.

### In vitro cytotoxicity evaluation

The methylthiazoltetrazolium (MTT) assay was used to test the in vitro anti-tumor activity of the formulation. 4T1 cells were seeded at a density of 1 × 10^4^ cells on 96-well plates and incubated at 37 °C overnight. Different formation was added into transwell inserts and incubated for another 48 h. Next, the transwell inserts and the medium were removed. The cells in each well were added with 20 µL of MTT solution and incubated for 4 h. Finally, the solution was replaced by 200 µL of DMSO to dissolve the purple formazan crystal. We applied a microplate reader to detect the absorbance of each well at 570 nm, and the assays were repeated for three times. The cell viability and combination index (CI) were calculated by Eq. (4) and Eq. (5), respectively.

4 $$\mathrm{Cell}\;\mathrm{viability}\;\%\;=\;\lbrack\;({\mathrm A}_{\mathrm S}-{\mathrm A}_{\mathrm B})/({\mathrm A}_{\mathrm C}-{\mathrm A}_{\mathrm B})\rbrack\times100\%$$

where A_S_, A_B_ and A_C_ were represented as the absorbance of sample treated group, control group and blank group, respectively.

5 $$\mathrm{CI}\;=\;{\mathrm D}_{\mathrm{DOX}}/{\mathrm D}_{50\mathrm{DOX}}+{\mathrm D}_{\mathrm{Rg}3}/{\mathrm D}_{50\mathrm{Rg}3}$$

where D_DOX_ and D_Rg3_ were the weight of DOX and Rg3 used in the combination group to achieve 50% inhibitory effect. D_50DOX_ and D_50Rg3_ were the weight of DOX and Rg3 to achieve 50% inhibitory alone.

### ICD effect evaluation: CRT exposure and release of ATP and HMGB1

To investigate CRT translocation, 4T1 cells were incubated with PBS, free Rg3, free DOX, Rg3 + DOX, Rg3-NPs + DOX, and Rg3-PNPs + DOX for 24 h, using 0.2 µg/mL DOX and Rg3. The cells were washed by PBS and incubated with rabbit anti-calreticulin (CRT) antibody for 30 min, washed with PBS and then incubated with anti-Rabbit IgG secondary antibody [26]. After this, the cells were fixed with 4% paraformaldehyde for 10 min, then stained with DAPI at 10 µg/mL and observed under the inverted confocal microscopy. Supernatants of 4T1 cells were collected to analyze ATP and HMBG1 levels according to the manufacturer’s instructions [27]. HMGB1 ELISA kit was purchased from Life Span BioSciences, and OD value was measured with a Spectra Maxmicroplate reader (Molecular Devices, USA). An enhanced ATP assay kit was obtained from Beyotime Biotechnology (Shanghai, China). ATP luminescence was detected by EnVision microplate reader (PerkinElmer, USA).

For in vivo ICD observation, 1 × 10^6^ 4T1 cells were injected to the right breast fat pad in female BALB/c mice. Mice were treated as indicated. At the end of the experiments, the tumors were collected and examined for ICD markers (HMGB1 and CRT) by immunohistochemical analysis. Primary antibodies CRT (ab92516), HMGB1 (ab79823) were purchased from Abcam.

### Tumor spheroid penetration assays

In order to model a 3D tumor environment, 3D breast cancer stroma-rich spheroids were prepared by co-culturing 4T1 breast cancer cells and NIH-3T3 fibroblast cells at the ratio of 1:2 according to the literature with modification [28]. By the handing drop method, spheroids formed with an average diameter of 500 ± 50 μm after 7 days of incubation. We utilized coumarin-6 (CUR) in place of Rg3 to perform the tumor spheroid penetration arrays. CUR, CUR-NPs, CUR-PNPs, CUR@PPP, CUR-NPs@PPP and CUR-PNPs@PPP were added into the plates, respectively and the incubation sustained for 24 h. Subsequently, collected tumor spheroid were washed with PBS for three times and fixed in 4% formaldehyde. Finally, the photos were collected by an inverted fluorescence microscope.

### Mitochondrial targeting assays

Briefly, 4T1 cells were seeded in a 12-well plate cultured overnight and then subjected to CUR, CUR-NPs, CUR-PNPs, CUR@PPP, CUR-NPs@PPP and CUR-PNPs@PPP for 4 h, respectively. The residual substances on the cell surface were washed by PBS thrice and then stained with MitoTracker Red solution (Beyotime Biotechnology, 25 nM) to stain the mitochondria of 4T1 for 30 min [29], and the cells were then washed by PBS and observed by confocal laser scanning microscope (CLSM).

### Animal feeding and management

Female BALB/c mice (6–8 weeks old, 18–20 g) were obtained from Shanghai SLAC Laboratory Animal Co., Ltd (Shanghai, China). Mice were fed with standard laboratory food and water, and received 12 h of light per day. All experiments on mice were performed under the guidelines approved by the Institutional Animal Care and Use Committee (IACUC) of Jiangsu Provincial Academy of Chinese Medicine with the approval number AEWC-20210426-139.

### Orthotopic breast tumor model for evaluation of Rg3-PNPs + DOX@PPP therapy

10^6^ 4T1 cells were injected into the right mammary fat pad of BALB/c mice at day − 7. When the tumor volume reached 100–200 mm^3^, the mice were randomly separated into six groups (n = 5) and received the treatments with PBS, Rg3@PPP, DOX@PPP, Rg3 + DOX@PPP, Rg3-NPs + DOX@PPP, and Rg3-PNPs + DOX@PPP, respectively. To determine whether Rg3 could boost DOX-induced ICD in vivo, tumor-bearing mice were peritumorally injected with PPP encapsulating DOX and/or Rg3 (4 mg/kg DOX and Rg3 equivalent) on days 1, 4, 8 and 11. Afterwards, mice were sacrificed on day 15. Tumor-draining lymph nodes were collected to analyze DC maturation by flow cytometry. The tumors were harvested for immunofluorescence and immunohistochemistry assays [30].

### DC maturation and antigens presentation ability evaluation ***in vivo***

Tumor-draining lymph nodes were mechanically passed through a 200-mesh filter. Nonspecific labeling was blocked with anti-mouse CD16/32 (Fc block, BioLegend, USA). DC maturation was examined by staining with Fixable Viability Dye eFluo 780 (eBioscience, USA), anti-CD45-Percy-cy5, anti-CD11c-APC, anti-CD80-PE, anti-CD86-BV421, and anti-MHCII-FITC antibodies, or an isotype IgG control (BioLegend, USA) [31]. For each sample at least 10^4^ cells were detected on a BD FACS Verse Flow Cytometer (BD, USA). Data obtained were analyzed using FlowJo 7.6 software (Tree Star, USA).

### Antitumor evaluation of Rg3-PNPs + DOX@PPP in combination with αPD-L1 in mice bearing unilateral breast cancer in situ

Female BALB/c mice (6–8 weeks old) were anesthetized and 10^6^ 4T1-luc cells were injected into the right mammary fat pad of BALB/c mice at day − 7. When the tumor volume reached 100–200 mm^3^, the mice were randomly separated into eight groups (n = 5) and received the treatments with PBS, PPP, Rg3@PPP, DOX@PPP, Rg3 + DOX@PPP, Rg3-PNPs + DOX@PPP, αPD-L1 and Rg3-PNPs + DOX@PPP + αPD-L1, respectively. Hydrogel PPP groups (4 mg/kg DOX or/and 4 mg/kg Rg3 equivalent) were peritumorally administrated four times, at day 1, day 4, day 8, and day 11. αPD-L1 (1.2 mg/kg) monotherapy or in combination with Rg3-PNPs + DOX@PPP, was administrated via tail vein four times, at day 1, day 4, day 8, and day 11. The tumor was measured by digital caliper, and was calculated as 0.5 × length × width^2^ (mm^3^). The tumor volume and body weight were monitored. Moreover, mice were subjected to IVIS optical imaging system (Perkin Elmer) on day 7, 11 and 15. On day 15, mice were sacrificed and the major organs (kidney, lung, spleen, liver, and heart) were assessed by H&E staining. The tumors were collected for immunohistochemistry and H&E staining. Tumor-draining lymph nodes were harvested to analyze T cells by flow cytometry. The survival curves were drawn according to mice survival percentages with time elapse.

### Establishment of the bilateral 4T1 tumor model to examine the abscopal effect

For the primary tumor inoculation, 10^6^ 4T1 cells were injected into the right breast fat pad of female BALB/c mice at day − 7. For the distal tumor inoculation, 5 × 10^5^ 4T1 cells were injected into the left breast fat pad at day − 3 [7]. After the primary tumor volume reached about 100-200mm^3^, mice were divided into six groups (*n* = 5): PBS, Rg3@PPP, DOX@PPP, Rg3-PNPs + DOX@PPP, αPD-L1@PPP (αPD-L1 loaded hydrogel) and (Rg3-PNPs + DOX + αPD-L1)@PPP. The dose of αPD-L1 was 1.2 mg/kg, and the dose of DOX and Rg3 was 4 mg/kg equivalent. Therapy was carried out only on the primary tumors. For in vivo combination therapies, αPD-L1 was also encapsulated into hydrogel with Rg3-PNPs + DOX. All hydrogel groups were peritumorally injected four times, at day 1, day 4, day 8, and day 11. The tumor volume of both the primary and the distant tumor were monitored accordingly. On day 15, the spleen and the distant tumors were collected to explore the ratio of memory T cells.

### Analysis of T cells

Tumors were surgically removed, mechanically cut into small pieces, and then digested with DNase (Thermo Scientific, USA), hyaluronidase (Gibco, USA) and collagenase IV (Gibco, USA) for 40 min at 37 °C. Tumor infiltrating lymphocytes (TILs) were isolated by single-step density gradient centrifugation in Percoll (GE Healthcare Bio-Sciences AB, Uppsala, Sweden). Tumor-draining lymph node and the spleen were mechanically passed through a 200-mesh filter. Red blood cells in the spleen were removed using ACK lysis buffer. Single-cell suspensions were stained with Fixable Viability Dye eFluor 780 (eBioscience, USA), anti-CD45-Percy-cy5, anti-CD4-APC, anti-CD8-FITC, and anti-IFN-γ-PE antibodies (Biolegend, USA) to differentiate cytotoxic T lymphocytes (CTLs, CD4^−^CD8^+^IFN-γ^+^) and TH1 cells (CD4^+^CD8^−^IFN-γ^+^). For intracellular staining, cells were stimulated for 6 h with Cell Activation Cocktail (with Brefeldin A) (Biolegend, USA). To detect regulatory T cells (Treg cells, CD4^+^Foxp3^+^) ratios, a Foxp3 Fixation and Permeabilization Kit were used as well as the anti-FoxP3-PE antibody (BD Biosciences, USA).

To examine the ratio of Tems (CD3^+^CD8^+^CD44^+^CD62L^−^), the lymphocytes were isolated from the abscopal tumors and the spleen, and stained with Fixable Viability Dye eFluo 780, anti-CD3-PE-Cy7, anti-CD4-APC, anti-CD8-FITC, anti-CD45-Percy-cy5, anti-CD62L-PE, and anti-CD44-BV421 antibodies (Biolegend, USA) for 20 min at 4 °C in the dark [18]. Afterwards, the cells were re-suspended with 150 µL PBS and detected by flow cytometry.

### Immunohistochemical staining and immunofluorescence evaluation

IHC staining was performed on mice tumor tissue Sect. (5 µM), followed by peroxidase blocking, blocking with 3% BSA, and primary antibodies incubation. Primary antibodies Ki67 (ab15580), CCL2 (ab7202), α-SMA (ab5694) were purchased from Abcam. Afterwards, slides were stained with HRP-labeled secondary antibody and hematoxylin counterstain.

Immunofluorescence for dual-staining of CD11c/CD86 was also conducted based on Tyramide Signal Amplification (TSA) method [32]. In brief, the tumor was first stained with anti-CD11c antibody (ab254183, Abcam) after antigen retrieval. After incubating with secondary antibody anti-mouse IgG H&L (HRP) (ab6728, Abcam), the sides were exposed to cyanine 3 tyramide to amplify fluorescein signal (red fluorescence). The sides were further incubated with anti-CD86 antibody (E5W6H, Cell Signaling Technologies) and then stained with FITC-labeled anti-rabbit IgG (green fluorescence) (ab6717, Abcam) with nuclear staining by mounting in medium containing DAPI (Vector Laboratories). For PD-L1 and CD8 staining, sides were incubated with primary antibodies at 4 °C overnight and then incubated with fluorescent secondary antibodies Goat Anti-Rabbit IgG H&L (Alexa Fluor® 647) (ab150079, Abcam) at 37 °C for 1 h and mounted with DAPI. All stained slides were visualized for three times under microscope.

### Statistical analyses

All experiments were performed at least three times and data were expressed as means ± SD. For individual comparisons, one-way ANOVA and a two-tailed, unpaired Student’s *t*-test were performed using GraphPad Prism 8.0 (GraphPad Software, USA). *P* < 0.05 was considered statistically significant.

## Results and discussion

### Synthesis and characterization of R_6_F_3_-CS

Peptides-Modified Chitosan (R_6_F_3_-CS) was synthesized by conjugating R_6_F_3_ to CS via an amide bond (Fig. S1). The successful synthesis of R_6_F_3_-CS was confirmed using FT-IR. As shown in Fig. S2a, the CS showed a distinctive absorption band at 3436.1 cm^− 1^ (the combination of stretching of –OH and –HN), and other absorption band at 2894.3 cm^− 1^, 1624.5 cm^− 1^ and 1517.0 cm^− 1^ were due to the stretching vibrational bands of –CH aliphatic, –C = O acetyl and the bending vibrational band of –NH, respectively. Compared with CS, the characteristic bands of R_6_F_3_-CS appeared at 1658.1 cm^− 1^ and 1553.3 cm^− 1^, which might be attributed to the amide Ι and amide II. Besides, the absorption peak of –CH_2_ at 2927.0 cm^− 1^ of R_6_F_3_-CS was much stronger than the peak of CS because the grafted CS increased the ratio of the methylene groups. Above results suggested that the R_6_F_3_ had been successfully grafted on the CS.

The successful introduction of R_6_F_3_ onto CS was also explored by ^1^HNMR spectroscopy. As shown in Fig. S2b and S2c, non-modified CS exhibited chemical shift at 3.16 to 4.89 ppm, which are probably ascribed to the glucosamine unit. The typical peak of R_6_F_3_ was detected at about 7.1 ppm for the amide bond. Moreover, some new peaks were observed at 1.1–1.5 ppm, also indicating that R_6_F_3_ had grafted to the backbone of CS. Afterwards, the nitrogen content was analyzed and presented in Table 1. The nitrogen content in CS was 8.65%, which was consisted with the theoretical value (8.7%). After conjugated with the peptides, the nitrogen content was obviously increased and reached to 16.32%. The grafting rate of peptides was 8.17% in the PEP-CS according to the Eq. (1), indicating that the peptides had been grafted onto CS.

**Table 1:** Grafting rate of peptides in PEP-CS

Polymers	Elemental analyse (Nitrogen content%)	Grafting rate (%)
CS	8.65 ± 0.13	/
PEP-CS	16.32 ± 0.21	8.17 ± 0.10

### Design of Rg3-loaded nanoparticle platform (Rg3-PNPs)

**Fig. 1:**
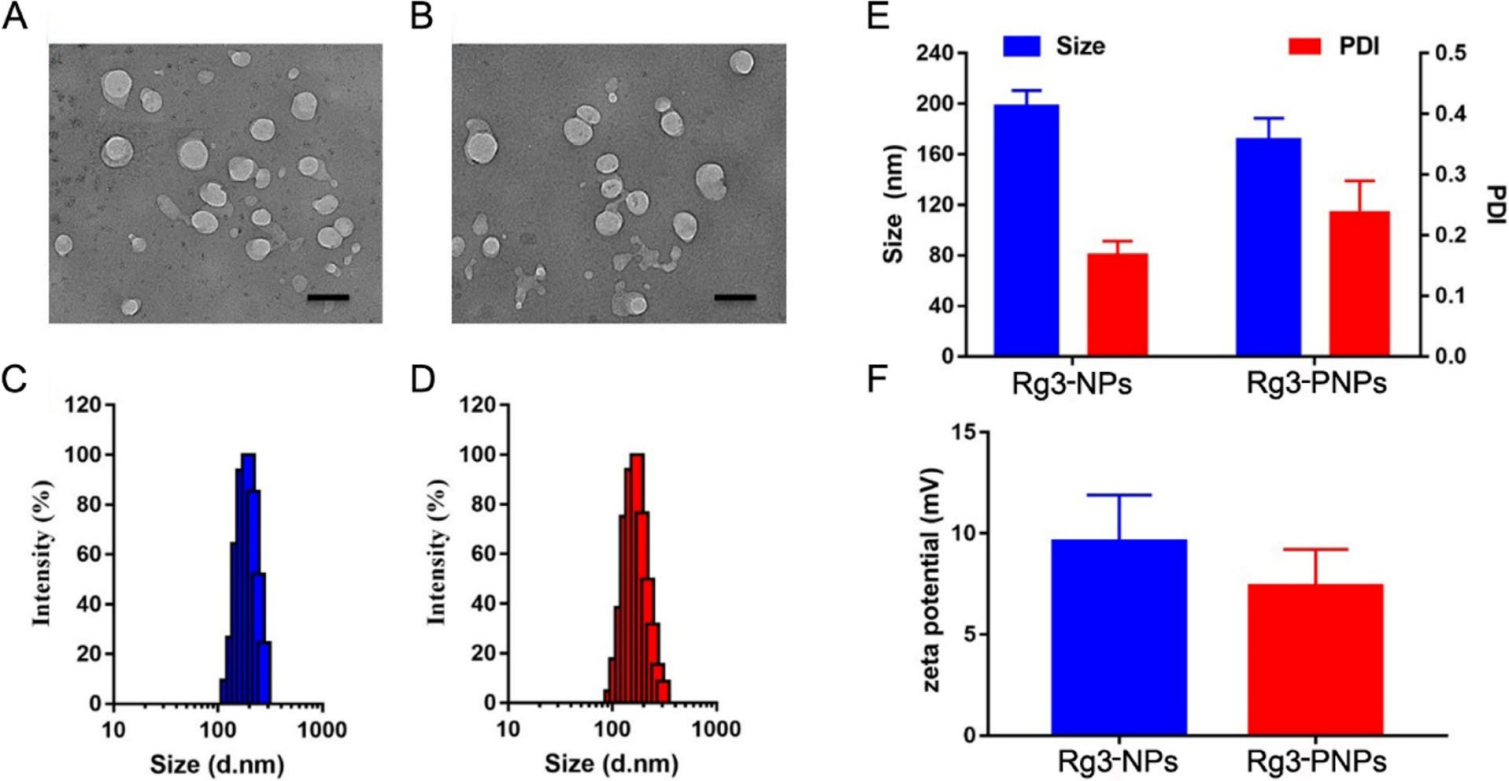
Preparation and characterization of Rg3-loaded nanoparticles. TEM images of (**A**) Rg3-NPs and (**B**) Rg3-PNPs, scale bar = 200 nm, particle size of (**C**) Rg3-NPs and (**D**) Rg3-PNPs, (**E**) size and PDI of Rg3-NPs and Rg3-PNPs, (**F**) zeta potential of Rg3-NPs and Rg3-PNPs

Rg3 has a great potential for the treatment of cancer, but the poor solubility of Rg3 in aqueous solution has limited its use in vivo, therefore, encapsulation of Rg3 into nanoparticle was developed in this research with the purpose of improving treatment efficiency. In this study, we utilized the CS and R_6_F_3_-CS to encapsulate Rg3 respectively, namely Rg3-NPs and Rg3-PNPs. Rg3-NPs and Rg3-PNPs presented a micelle-like structure with a diameter of approximately 200 nm. In detail, the particle size for Rg3-NPs and Rg3-PNPs was about 198 nm and 171 nm, respectively (Fig. 1C). Because the amphiphilic cell-penetrating peptides (CPPs) promote the self-assembly of nanoparticles, a decline in size was observed after surface modifications with CPPs. The zeta potential of Rg3-NPs and Rg3-PNPs was about 9.7 mV and 8.6 mV, as confirmed by dynamic light scattering. And the PDI of Rg3-NPs and Rg3-PNPs were only 0.18 and 0.23, inferring that the nanoparticle had narrow size distribution and is appropriate for passive targeting by the EPR effect. Furthermore, Rg3-NPs and Rg3-PNPs exhibited a similar size after storage in the aqueous suspension for 7 days, suggesting the excellent stability (Fig. S3). The HPLC analysis showed that the drug loading (DL) of Rg3 was 8.7 ± 1.2% and 9.2 ± 1.7% in Rg3-NPs and Rg3-PNPs, respectively (Tab. S1). Above date indicated that the Rg3-NPs and Rg3-PNPs are suitable formulation for the delivery of Rg3 in tumor treatment.

### Rg3-PNPs promoted cytotoxicity and ICD-inducing effect of DOX in vitro

DOX is a representative chemotherapeutic agent which is widely used in the treatment of breast cancer. In this research, we attempted to combine Rg3 with DOX to verify whether the synergistic effect would work. In order to verify the optimal anti-tumor ratio between DOX and Rg3, MTT assays were conducted in this research. All groups exhibited the dose-dependent cytotoxicity after 48 h incubation, the lower IC50 value and all groups of CI value < 1 was observed after combination (Fig. S5), the strongest synergistic result was achieved when the weight ratio of DOX and Rg3 was 1:1 with the CI value of 0.44, and the IC50 of DOX and Rg3 decreased to 1.32 µg/mL at this ratio compared to 17.41 µg/mL of Rg3 and 3.64 µg/mL of DOX, respectively. Herein, the combination of DOX and Rg3 with the weight ratio of 1:1 was chosen for the further research.

**Fig. 2:**
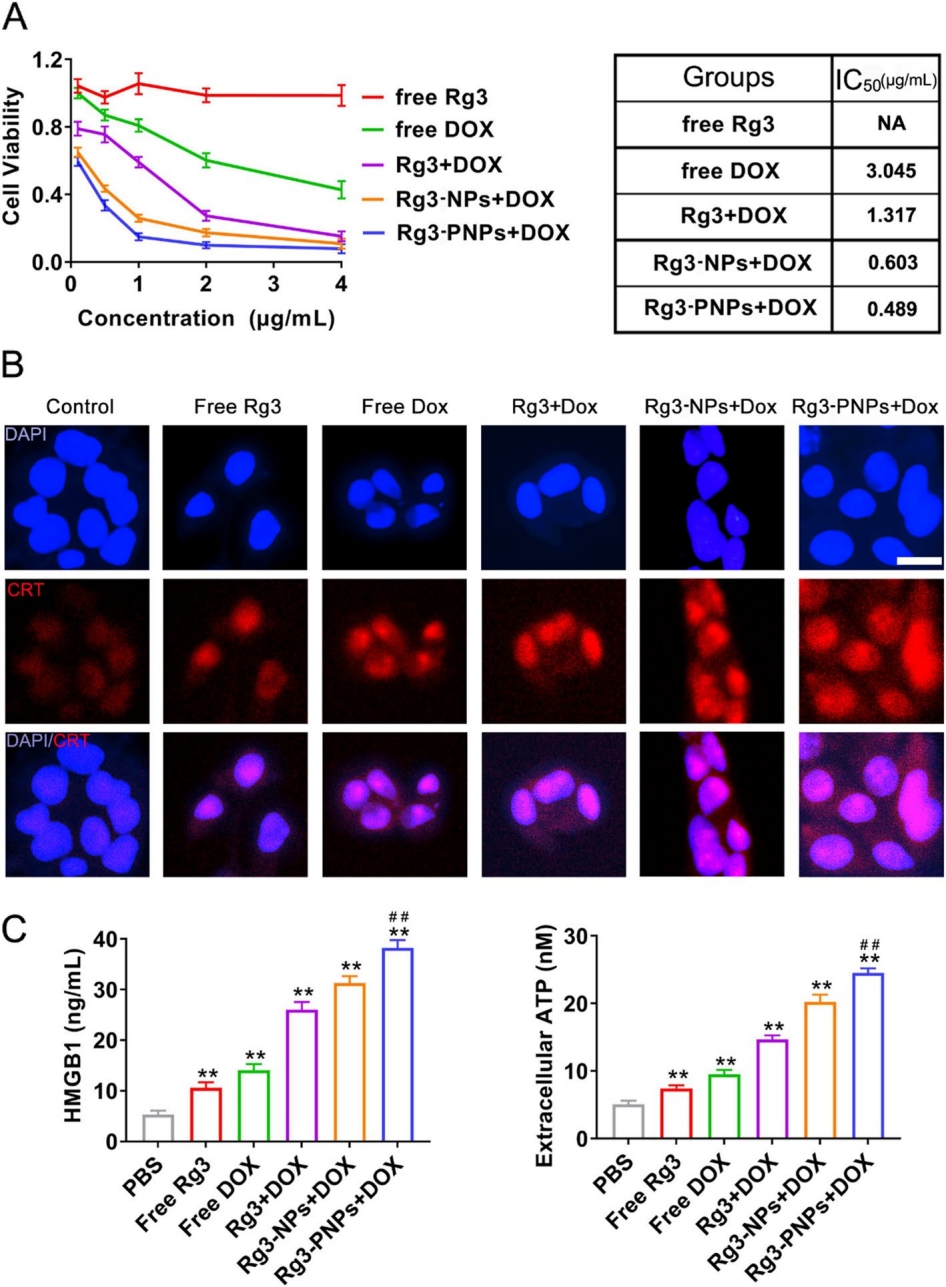
Rg3-PNPs promoted the immunogenic cell death of 4T1 induced by DOX. **A** Cytotoxicity of different group against 4T1 cells after 48 h incubation and the corresponding IC_50_ values. **B** Immunofluorescence staining of CRT expression on 4T1 cells after 24 h incubation. **C** HMBG1 and extracellular ATP levels in the supernatants of 4T1 cells after 24 h incubation. ^*^p < 0.05 and ^**^p < 0.01 vs. the PBS group. ^##^ *p* < 0.01 vs. the free DOX group

The in vitro cytotoxicity of synergistic effect between Rg3 nanoparticles and DOX was also evaluated. As shown in Fig. 2A, Rg3 at test concentrations displayed no obvious inhibition on 4T1 cells. Free DOX inhibited the 4T1 cell growth in a concentration-dependent manner, while the anti-tumor effect was augmented after the addition of Rg3. The preparation of nanoparticles further strengthened the antitumor effect of Rg3. The IC_50_ of free DOX, Rg3 + DOX, Rg3-NPs + DOX and Rg3-PNPs + DOX were 3.045, 1.317, 0.603 and 0.489 µg/mL, respectively. Nanoparticles encapsulating R_6_F_3_-CS modified Rg3 (Rg3-PNPs) achieved the strongest antitumor efficacy in combination with DOX, with the IC_50_ value of DOX dropping from 3.045 µg/mL to 0.489 µg/mL. In summary, Rg3 improved the antitumor efficacy of DOX, which was further reinforced by R_6_F_3_-CS modification and nanoparticles encapsulation.

Accumulating studies have documented that DOX can induce ICD and stimulate immune responses against tumor cells. However, DOX suffer from insufficient DAMPs to reach satisfied antitumor immune response by itself [15]. To measure whether Rg3 was able to strengthen ICD induced by DOX, we examined a range of DAMPs as the indicators of ICD generation, which include CRT exposure, the level of ATP and HMGB1. During the pre-apoptotic death process, CRT is exposed on cell membrane and activates DCs to phagocytize dead tumor cells. As shown in immunofluorescence analysis, 4T1 cell in the Rg3 group displayed negligible CRT exposure (Fig. 2B). Compared to DOX group, the Rg3 + DOX group exhibited a modest enhancement of CRT exposure, indicating Rg3 could improve the ICD effect induced by DOX. It is noteworthy that a dramatic increment of CRT translocation from lumen to cell membrane in Rg3-PNPs + DOX group, in which Rg3 was modified with R_6_F_3_-CS.

Encouraged by the above results, two other ICD biomarkers, HMGB1 and ATP, were also assessed. HMGB1 is a nuclear cytokine, secreted by the cells undergoing ICD. HMGB1 can initiate T cell-mediated immune responses [33]. ATP attracts macrophages and DCs into the tumor site [34]. HMGB1 level rose from 5.3 ± 0.8 ng/mL in PBS group to 14.1 ± 1.2 ng/mL in 4T1 cells supernatant of DOX group (Fig. 2C). HMGB1 release and ATP secretion in the Rg3-PNPs + DOX group was significantly higher than DOX group (*p* < 0.01). To sum up, the combination of Rg3-loaded nanoparticles and DOX considerably heightened ATP secretion, HMGB1 release and CRT exposure, which might hold a great potential in immune-based therapy.

### Design and characteristic of the hydrogel platform (PPP) for DOX and Rg3 co-delivery

**Fig. 3:**
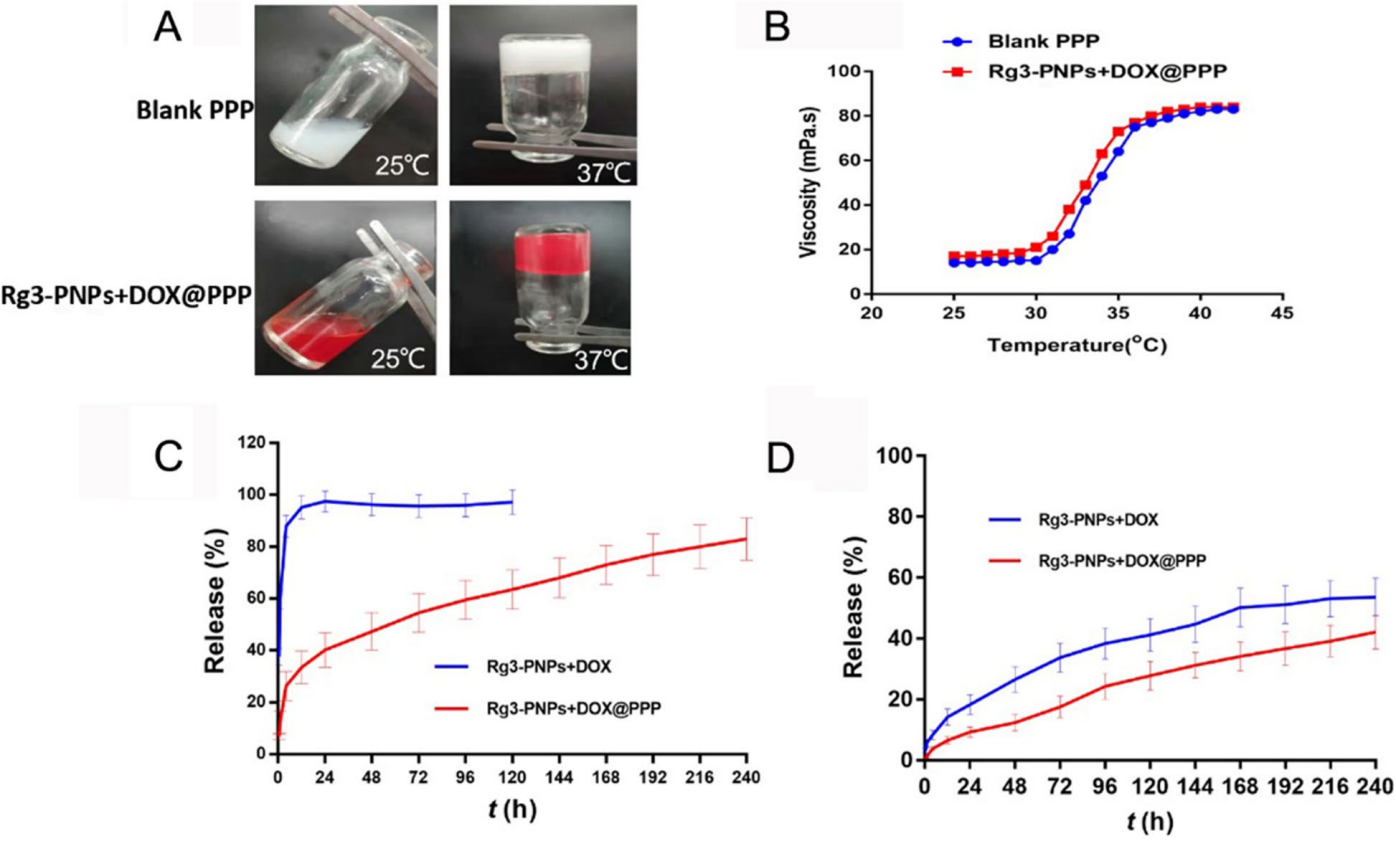
Preparation and characterization of hydrogel formulation (PPP) encapsulating Rg3-PNPs and DOX. **A** Blank PPP and Rg3-PNPs + DOX@PPP were in a sol state at room temperature (25 °C) and transformed into the solid gel at 37 °C. **B** Viscosity analysis of blank PPP and Rg3-PNPs + DOX@PPP with respect to temperature. **C** In vitro release curve of DOX from Rg3-PNPs + DOX and Rg3-PNPs + DOX@PPP. **D** In vitro release curve of Rg3 from Rg3-PNPs + DOX and Rg3-PNPs + DOX@PPP.

The development of traditional tumor-targeted delivery system was still based on receptor-mediated active delivery and EPR effect-mediated passive targeting delivery [35]. Although the combination of Rg3-loaded nanoparticle and free DOX achieved enhanced anti-tumor effect, quick elimination of the reticuloendothelial system often resulted in low targeting efficacy in vivo. Local chemotherapy is capable of maximizing the concentration of drugs in tumor site and decreasing the side effect. Thermo-sensitive hydrogel like PLGA-PEG-PLGA (PPP) has been widely used for cancer treatment [36, 37]. As shown in Fig. 3A, both blank PPP and Rg3-PNPs + DOX@PPP were in liquid form at the room temperature, and the status gradually changed and converted into gel state when the temperature rose to 37 °C. The viscosity of blank PPP and Rg3-PNPs + DOX@PPP with respect to temperature was shown in Fig. 3B. Both of them showed moderately low viscosity below 30 °C, which provided the convenience for injection at the room temperature. However, the viscosity increased sharply when the temperature reached 38 °C, indicating the transition from sol to gel.

The in vitro release of Rg3-PNPs + DOX and Rg3-PNPs + DOX@PPP were performed under physiological conditions (37 °C, pH 7.4). As shown in Fig. 3C and D, the drug release profile of Rg3-PNPs + DOX showed the initial burst release of DOX, and over 80% of DOX was released within 6 h. Meanwhile, much slower DOX release was observed in Rg3-PNPs + DOX@PPP group, with only 30% release of DOX during the initial 12 h. And continuous release kept until 240 h. The release profile of Rg3 exhibited the similar trend as DOX, after encapsulation into hydrogel. Likewise, continuous and sustained release of Rg3 was detected during the 240-h period. Taken together, the release of DOX and Rg3 from Rg3-PNPs + DOX@PPP exhibited a sustained co-delivery manner in 240 h. The continuous release could maintain higher drug concentration in the tumor site and thus improving the therapeutic effect. In addition, blank PPP showed the fine stability with the mean diameters of 30 nm and the zeta potential of -0.51 mV (Fig. S4 and Tab. S2), and the Rg3-PNPs + DOX@PPP exhibited a pleasant stability over 7 days. The mean diameters of Rg3-PNPs + DOX@PPP was around 220 nm, which might be caused by the electrical adsorption between the negative charge of PPP and the positive charge of Rg3-PNPs.

### PNPs@PPP formulation achieved a deep tumor penetration on multicellular spheroids

**Fig. 4:**
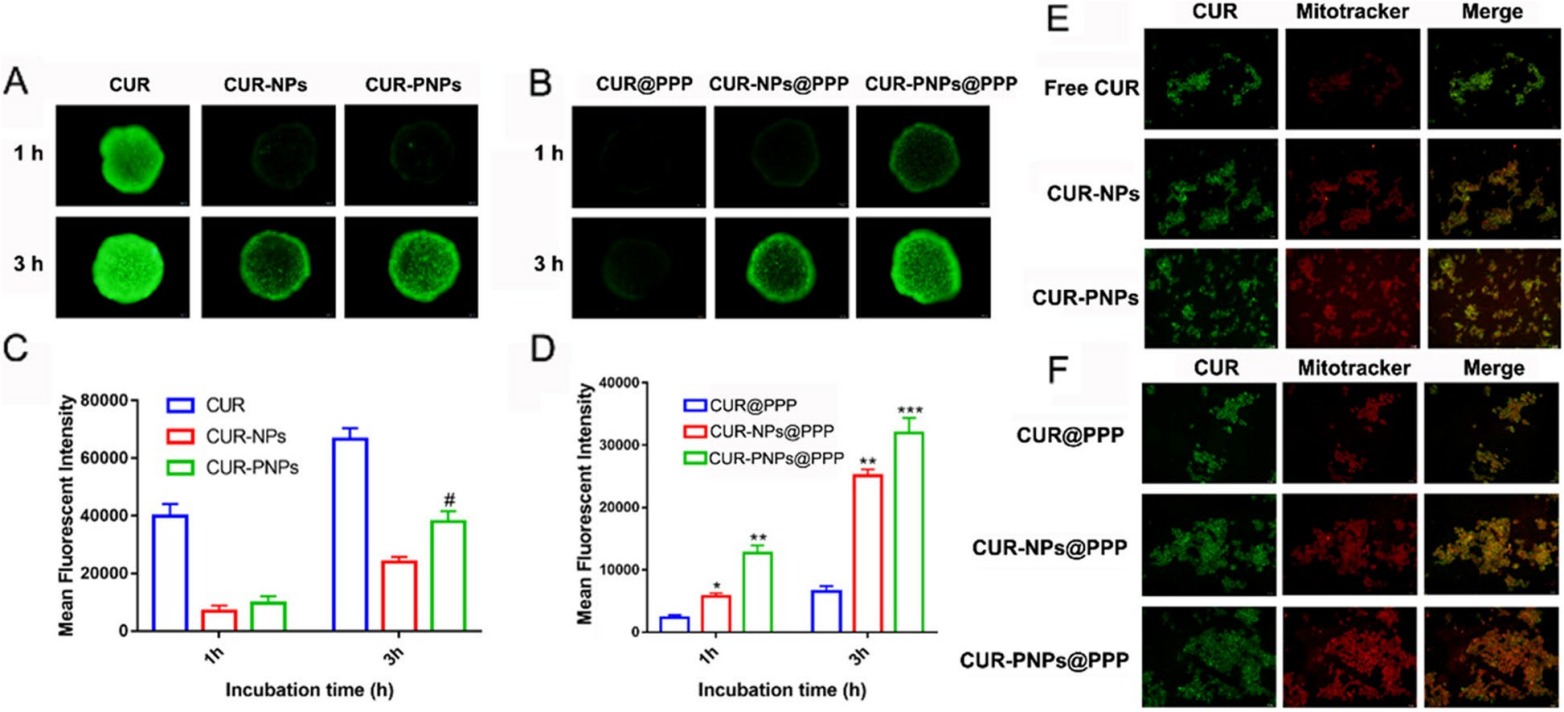
Tumor spheres penetration and mitochondrial co-localization of hydrogels in 4T1 cells. Representative images of uptake and penetration of (**A**) CUR-PNPs and (**B**) CUR-PNPs@PPP in tumor spheres. Quantitative analysis of the mean fluorescence intensity of (**C**) CUR-PNPs and (**D**) CUR-PNPs@PPP. Data represented as mean ± SD from at least 3 spheroids per condition. ^*^p < 0.05 and ^**^p < 0.01 vs. CUR, ^#^*p* < 0.05 vs. CUR-NPs. Co-localization of (**E**) CUR-PNPs and (**F**) CUR-PNPs@PPP into the mitochondria of 4T1 cells after 2 h incubation. Orange spots in the merged images denoted the co-localization within the mitochondria. Red: Mitotracker red. Green: CUR

In immune “cold” tumors, T lymphocytes infiltration is deserted at the tumor center. Therefore, chemotherapy with deep tumor penetration is needed to induce potent ICD, thus recruiting more T cells into the tumor center. Tumor spheroids are widely used to mimic tumor environment, including diffusion gradients, pathophysiological milieu conditions and tumor macrostructure [38]. Poor drug penetration can be attributed to the high interstitial fluid pressure [39]. To examine whether R_6_F_3_-CS modification could promote the permeability of PPP formulation, multicellular spheroids composed of activated NIH/3T3 and 4T1 cells were constructed with the diameter ranging from 450 to 550 μm. Coumarin-6 (CUR) was utilized to replace Rg3 to perform the tumor spheroid penetration arrays. CPPs with 6 arginines have been reported to induce trans-membrane transportation of the carrier, thus increasing the penetration ability of the PNPs [40]. As shown in Fig. 4A and B, free CUR group showed the strongest fluorescence intensity, which might be due to the easy penetration of small molecule into the tumor, but it is difficult to come true in vivo owing to the lack of targeting and the quick clearance by the endothelial reticular system. Meanwhile, the CUR@PPP showed weak fluorescence and was mainly located in the outer cell layers of tumor sphere after 1 and 3 h incubation, with a penetration depth of 80 μm at most. The fluorescence intensity of CUR@PPP at 1 and 3 h was only 2314.2 and 6523.1 respectively. The penetration efficacy of CUR-NPs@PPP and CUR-PNPs@PPP showed a 2.85- and 3.91-folds increase compared with CUR@PPP after 3 h treatment, and the fluorescence signal have been widely distributed, even reaching the center of the tumor spheroids. These results demonstrated that CS or R_6_F_3_-CS could deliver drugs deeply into tumor spheroids. Besides, due to the local injection of hydrogel, drugs elimination during the process of internal circulation is avoided to the greatest extent.

### PNPs@PPP formulation displayed mitochondrial targeting

As the vital cellular organelles, mitochondria play an essential role to produce energy, maintain metabolic homeostasis and are regarded as the critical regulators of cell death in apoptosis [41]. It has been reported that mitochondrial damage induced by drugs can evoke ICD [42], attributable to mitochondria dysfunction and subsequent amplification of oxidative stress [43]. Therefore, mitochondrial targeting has emerged as an attractive approach in cancer theranostics. In Fig. 4E, F, the orange fluorescence in the merged picture indicated the co-localization of the red signal from MitoTracker and the green signal from CUR. The Mito-tracker Red emitted red fluorescence and could locate to the mitochondria. The red fluorescence in CUR and CUR@PPP groups was relatively weak, meaning less localization of CUR to mitochondria. Comparatively, the merged images turn orange in CUR-NPs and CUR-PNPs groups, inferring CUR accumulation in mitochondria. Evidently, CUR-PNPs@PPP was localized in the mitochondria verified by the abundant orange signals. The results suggested that the CS or R_6_F_3_-CS with positive charges could improve electrostatic interaction with mitochondria of the tumor, allowing the drug targeted to the mitochondria.

### Rg3-PNPs + DOX@PPP promoted immunogenic cell death and DC maturation in mice bearing unilateral 4T1 tumor

**Fig. 5:**
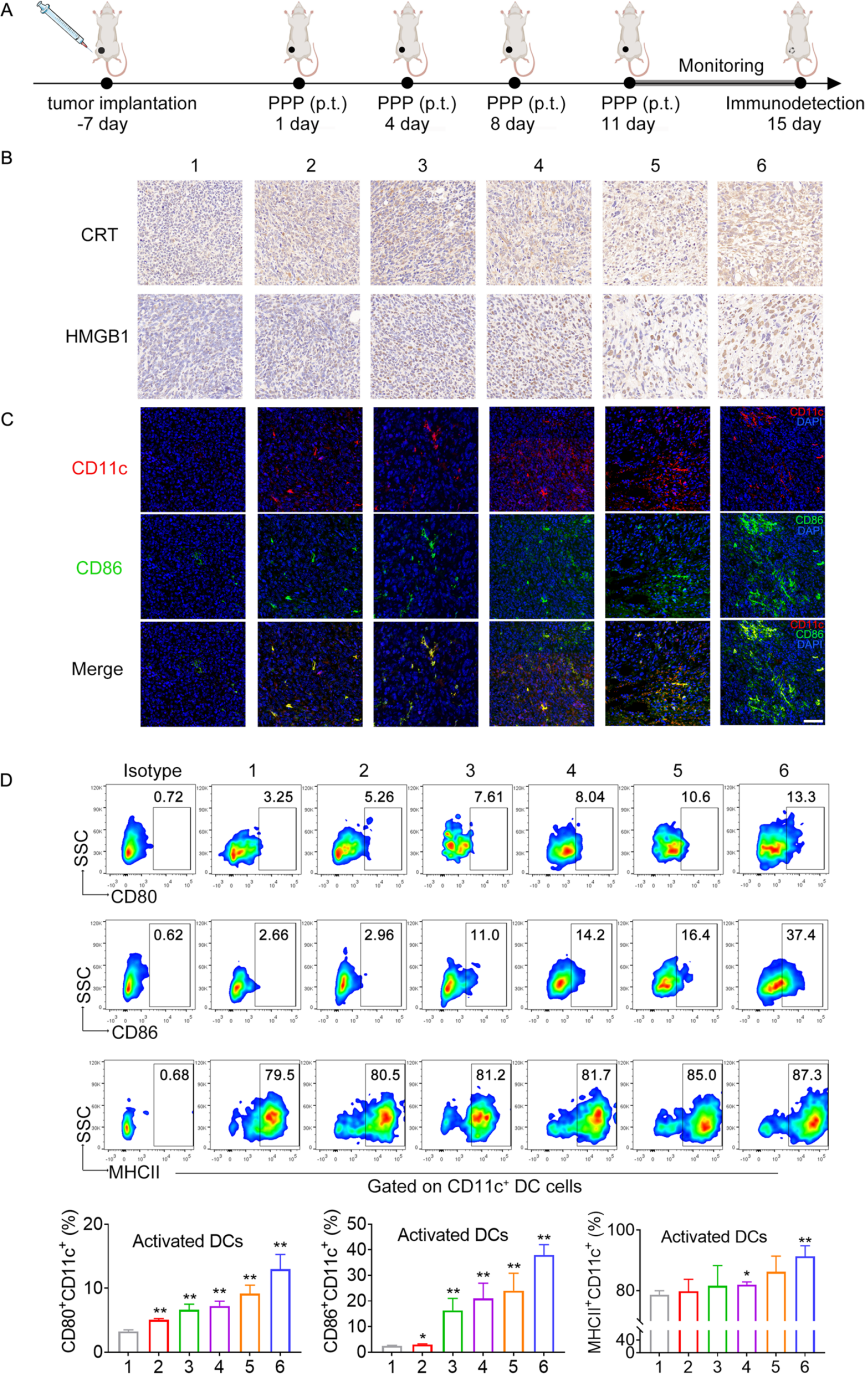
Rg3-PNPs@PPP and DOX combination promoted ICD effect and DC maturation in mice bearing 4T1 tumor. (**A**) Schedules for the indicated treatment. p.t. means peritumoral. (**B**) Immunohistochemistry of CRT and HMGB1 expression in tumors. (**C**) Immunofluorescence dual-staining of CD11 (red) and CD86 (green) in tumors sections. (**D**) Populations of CD80^+^, CD86^+^ and MHCII^+^ cells (matured dendritic cells markers), gated on CD11c^+^ DC cells in tumor-draining lymph nodes by flow cytometry. Data are presented as the mean ± SD (*n* = 3). 1. PBS; 2. Rg3@PPP; 3. DOX@PPP; 4. Rg3+DOX@PPP; 5. Rg3-NPs+DOX@PPP; 6. Rg3-PNPs+DOX@PPP. ^*^p < 0.05, ^**^p < 0.01 vs the PBS group

In order to investigate the ICD-inducing effect in vivo by Rg3-PNPs and DOX, immunohistochemical assays were performed to evaluate the expression of HMGB1 and CRT in tumor sections. Schedule for the indicated treatment was shown in Fig. 5A. The expression of HMGB1 and CRT was relatively low in the PBS group or Rg3 group. Meanwhile, CRT translocation to the cell membrane and HMGB1 expression significantly increased after Rg3-PNPs + DOX treatment, compared with DOX group. The results indicated the local chemotherapy could aggravate ICD to the greatest extent.

CRT translocates to cell membrane and emit the “eat me” sign to promote the phagocytosis of dead cells by DC, and HMGB1 facilitates the maturation of DC and the antigen presenting ability [44]. Mature DCs are the dominant antigen presenting cells in human body and is characteristic of MHCII, CD86 and CD80. Therefore, CD11 and CD86 expression was evaluated by immunofluorescence staining in tumor sections. The percentage of CD11 and CD86 double-positive cells in PBS group was the lowest among all groups. DOX has a slight effect on DC maturation, which could be enhanced by Rg3. Consistent with the ICD-inducing effect, samples from the Rg3-PNPs + DOX@PPP group had the most vigorous fluorescence intensity of CD11^+^ and CD86^+^ cells. We further analyzed the expression of CD11c^+^CD80^+^, CD11c^+^CD86^+^ and CD11c^+^MHCII^+^ (matured DC markers) in tumor draining lymph nodes by flow cytometry. The results showed that the Rg3-PNPs + DOX@PPP group induced the most robust acceleration in DC maturation in mice, causing the strongest immune response. Specifically, the CD11c^+^CD80^+^ DC cells in Rg3-PNPs + DOX@PPP group were 4.1-folds of the PBS group, and 1.7-folds of the DOX@PPP group, indicating that the formulation of locally peritumoral chemotherapy to co-deliver DOX and Rg3-PNPs could induce phenotypic maturation of DCs, thus remodeling the immune microenvironment to suppress breast cancer development.

### The combination of local chemotherapy and immunotherapy inhibited unilateral breast tumor growth in situ

**Fig. 6:**
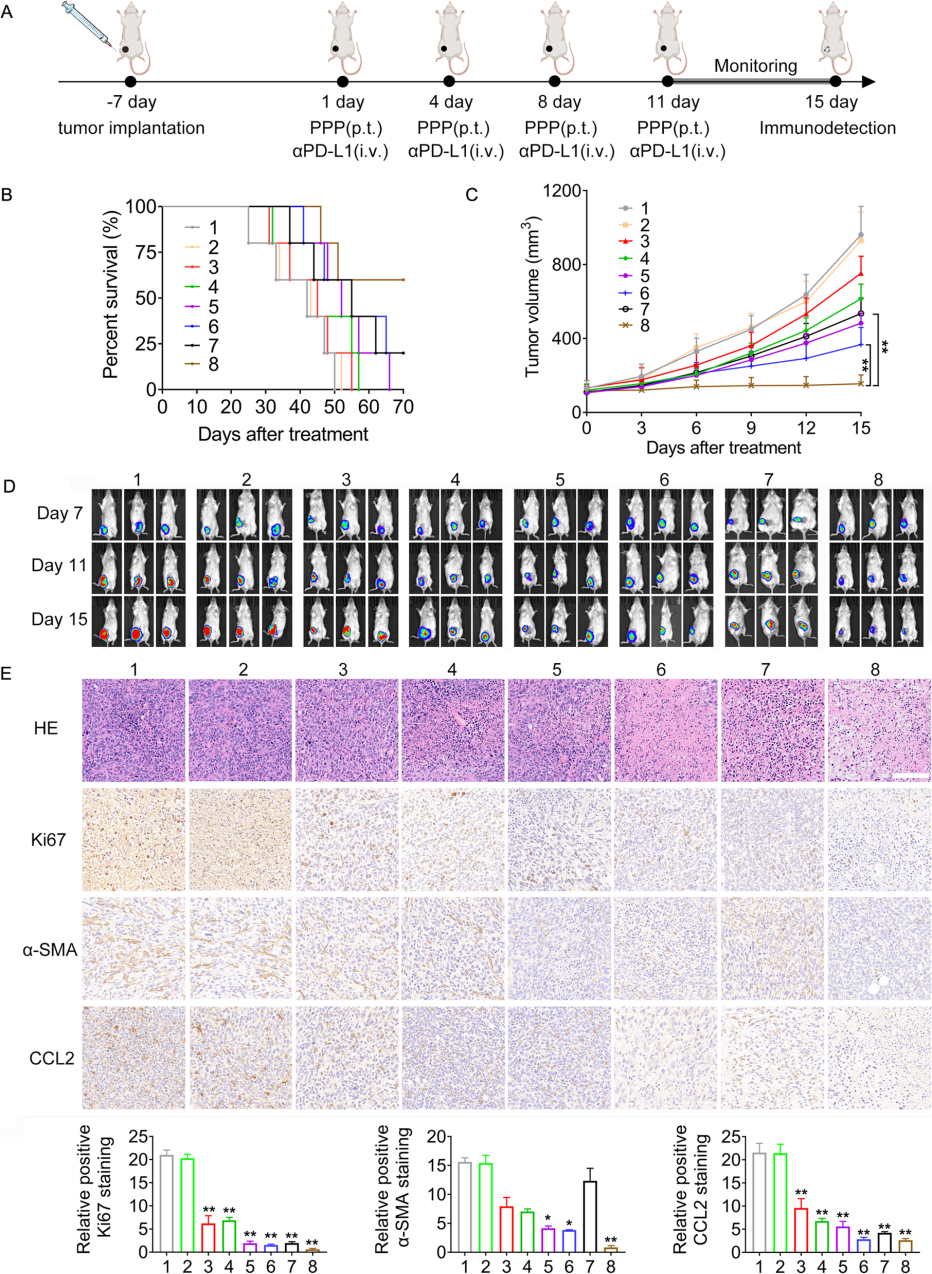
Antitumor efficiency of Rg3-PNPs + DOX combined with αPD-L1 in the orthotopic breast tumor model. **A** Schedules for the indicated treatment. p.t. means peritumoral. i.v. means intravenous. **B** Survival and (**C**) Tumor volume of each group (*n* = 5). **D** In vivo bioluminescence imaging of the 4T1-LUC tumors in different groups. **E** H&E staining and immunohistochemical analysis of Ki-67, α-SMA, CCL2 in tumor tissue (*n* = 3). (1) PBS; (2) PPP; (3) Rg3@PPP; (4) DOX@PPP; (5) Rg3 + DOX@PPP; (6) Rg3-PNPs + DOX@PPP; (7) αPD-L1; (8) Rg3-PNPs + DOX@PPP plus αPD-L1. ^*^p < 0.05, ^**^p < 0.01 vs. the PBS group

4T1 murine TNBC cell line shares genomic features of basal-like breast cancer, which is recognized as nonimmunogenic [45]. We established syngeneic 4T1 breast tumors orthotopically, which have been reported to have low immune response with insufficient immunogenicity [31], in order to investigate whether Rg3-PNPs + DOX@PPP can turn “cold tumor” into “hot tumor”. The schedule for the indicated treatment was shown in Fig. 6A. As expected, there was no distinction in tumor volume between PBS group and PPP vehicle group. From bioluminescence images, the DOX and Rg3 groups showed a weak therapeutic effect, similar to the PBS group (Fig. 6C, D). Both Rg3-NPs + DOX@PPP group and Rg3-PNPs + DOX@PPP group displayed tumor remission to a certain degree, indicating that the local chemotherapy benefits the therapeutic efficacy of DOX, but tumor growth was not sufficiently inhibited. αPD-L1 alone exerted a moderate, but not satisfactory effect on tumor growth, attributable to the “cold” properties of 4T1 tumor. In contrast, the best effect was achieved in the Rg3-PNPs + DOX@PPP + αPD-L1 group. Kaplan–Meier survival curve showed that Rg3@PPP or DOX@PPP group exhibited an unsatisfactory effect on mice survival (Fig. 6B). Mice treated with PBS were all dead within 50 days. 80% of mice died within 70 days in αPD-L1 group. In Rg3-PNPs + DOX@PPP + αPD-L1 group, 60% of mice survived over 70 days, which was meaningfully longer than other groups. In summary, the tumor volume and survival rate demonstrated an ideal synergistic performance in Rg3-PNPs + DOX@PPP plus αPD-L1 group.

H&E staining showed that tumor cells were densely arranged in the PBS group (Fig. 6E), with large and heteromorphic nuclei. Ruptured cells and chromatin condensation were observed in Rg3-PNPs + DOX@PPP group, suggesting the necrosis in tumor cells. Sparse arrangement, more gaps and large-area necrosis were found in the Rg3-PNPs + DOX@PPP + αPD-L1 group, signifying that the local chemotherapy and immunotherapy combination manifested more potent anti-cancer effect. High proliferative activity (Ki67 staining) was found in PBS group. The combination group showed the lowest proliferation index (Ki67).

As we know, the dense extracellular matrix (ECM) in the tumor microenvironment seriously limited the penetration of drugs within tumors [46]. Immune-cold tumors with remodeled stroma fail to respond to αPD-L1 monotherapy [47]. CCL2, also known as monocytic chemotactic protein 1 (MCP-1), was secreted by TME to promote tumor cells proliferation [48, 49]. The overexpression of CCL2 promotes tumor metastasis and invasion [50]. CCL2 has an unfavorable effect on prognosis in tumor patients because of the accumulation of immunosuppressive cell subtypes [51]. Both CCL2 and α-SMA expression was high in PBS group, indicating that the matrix proteins were abundant in tumors. Both Rg3 + DOX@PPP group and Rg3-PNPs + DOX@PPP group reduced α-SMA and CCL2 expression moderately. Over 60% elimination of α-SMA and CCL2 was observed in Rg3-PNPs + DOX@PPP + αPD-L1 group (Fig. 6E). The remodeling capability on ECM and small particle size contributed to the penetration of Rg3-PNPs, thus improving the anti-tumor effect.

### Changes in the tumor microenvironment following local chemotherapy and immunotherapy combination

**Fig. 7:**
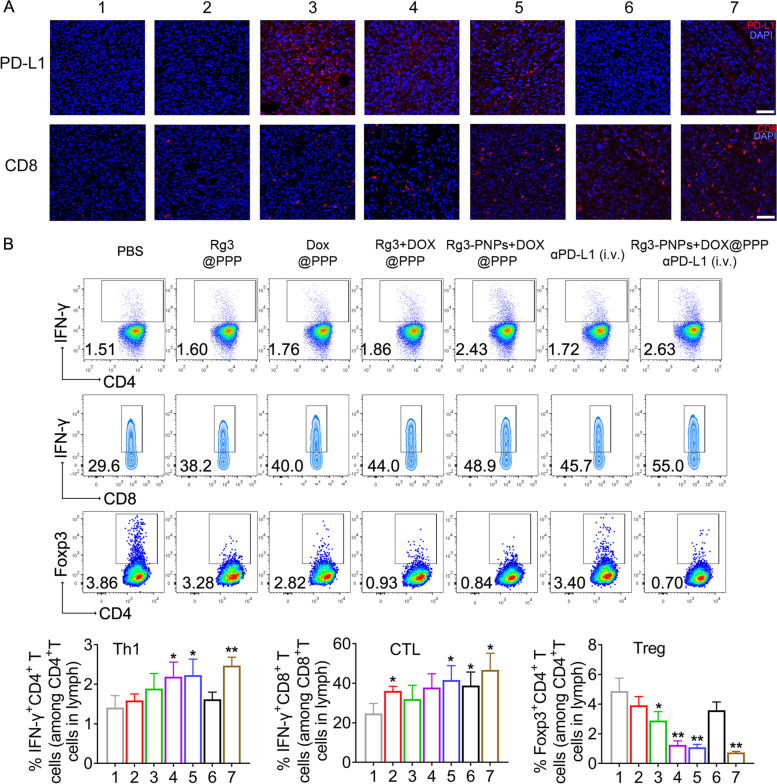
Combination of Rg3-PNPs+DOX with αPD-L1 activated T cells in mice bearing 4T1 tumor. (**A**) Immunofluorescent staining of CD8 and PD-L1 expression in tumors. (**B**) Flow cytometry analysis to show the level of TH1 (CD4^+^IFN-γ^+^ T), CTL (CD8^+^IFN-γ^+^ T) and Treg (CD4^+^Foxp3^+^ T) in tumor-draining lymph nodes. Error bars indicate SD (*n* = 3). 1. PBS; 2. Rg3@PPP; 3. DOX@PPP; 4. Rg3+DOX@PPP; 5. Rg3-PNPs+DOX@PPP; 6. αPD-L1 (i.v.); 7. Rg3-PNPs+DOX@PPP+αPD-L1 (i.v.). ^*^p < 0.05, ^**^p < 0.01 vs PBS group

Infiltrated lymphocytes closely reflect the immune status of the tumor microenvironment. Hence, T cells activation was assessed. Schedule for the indicated treatment was shown in Fig. 6A. Tumor antigen-specific cytotoxic T cells (CTL, CD8^+^IFN-γ^+^) which could bind to antigen MHCI complex, directly eradicate tumor cells [52]. 4T1 tumor has been regarded as immune-deserted ones [53], and immunofluorescent staining demonstrated that infiltration of CD8^+^ T cells was lacking in PBS-treated tumor (Fig. 7A). In Rg3 + DOX@PPP and Rg3-PNPs + DOX@PPP group, increment of CD8^+^ T cells infiltration was found, suggesting the transform of immune-deserted tumors into immune-cultivated ones. Rg3-PNPs + DOX@PPP plus αPD-L1 group stimulated the host immune response with the largest recruitment of CD8^+^ T cells into tumor tissues.

PD-L1 on tumor cells could bind to PD1 receptors on the T cell surface and caused T cell anergy. PD-L1 expression in tumors was also evaluated. Consistent with other research following chemotherapy [54], PD-L1 expression was adaptively augmented in tumors from DOX@PPP group (Fig. 7A). Rg3 has been regarded as a new agent targeting PD-L1 [55]. Therefore, PD-L1 expression was relatively lower in Rg3-PNPs + DOX@PPP group than DOX@PPP group. PD-L1 expression in tumors decreased sharply in Rg3-PNPs + DOX@PPP + αPD-L1 group, when compared with DOX@PPP group. Despite the ICD-induced immune response triggered by Rg3-PNPs + DOX, the anti-cancer efficacy of these groups was less satisfactory. Combination of immune checkpoint inhibitors with local chemotherapy has a potential in clinic therapy.

Helper TH1 cell (CD4^+^IFN-γ^+^) is a dominant effector cells against cancer, not only fostering the ability of NK and CTL cells but also provoking host immunity. Meanwhile, Treg induce an immunosuppressive state and limit the efficacy of chemotherapy. Therefore, we further analyzed the percentage of TH1, CTL and Treg in lymph nodes by flow cytometry. αPD-L1 monotherapy enlarged the CTL cell population in the lymph (Fig. 7B), but did not alter Treg and TH1 cells. The Rg3-PNPs + DOX@PPP plus αPD-L1 group had the largest proportion of CD8^+^ T cells in the lymph. Rg3-PNPs + DOX@PPP also promoted CTL and TH1 population, and inhibited Treg, but to a less extent than Rg3-PNPs + DOX@PPP + αPD-L1 group, which is consistent with the results from immunofluorescent staining. Above results showed the combo of the local chemotherapy and αPD-L1 could remodel the tumor microenvironment, and sensitize immunologically “cold” tumors for αPD-L1 treatment.

There was no loss of body weight among all treated groups (Fig. S7A). To further evaluate the safety of the formulation, we also utilized the H&E staining. The results showed that no significant histopathological observations on the major organs including heart, liver, spleen, lung, and kidney. Furthermore, no chronic inflammation was found in the skins after subcutaneous injection of thermogel (Fig. S6 and S7B), indicating that PPP possessed a good biocompatibility.

### Abscopal therapeutic effect investigations in bilateral tumor model

**Fig. 8:**
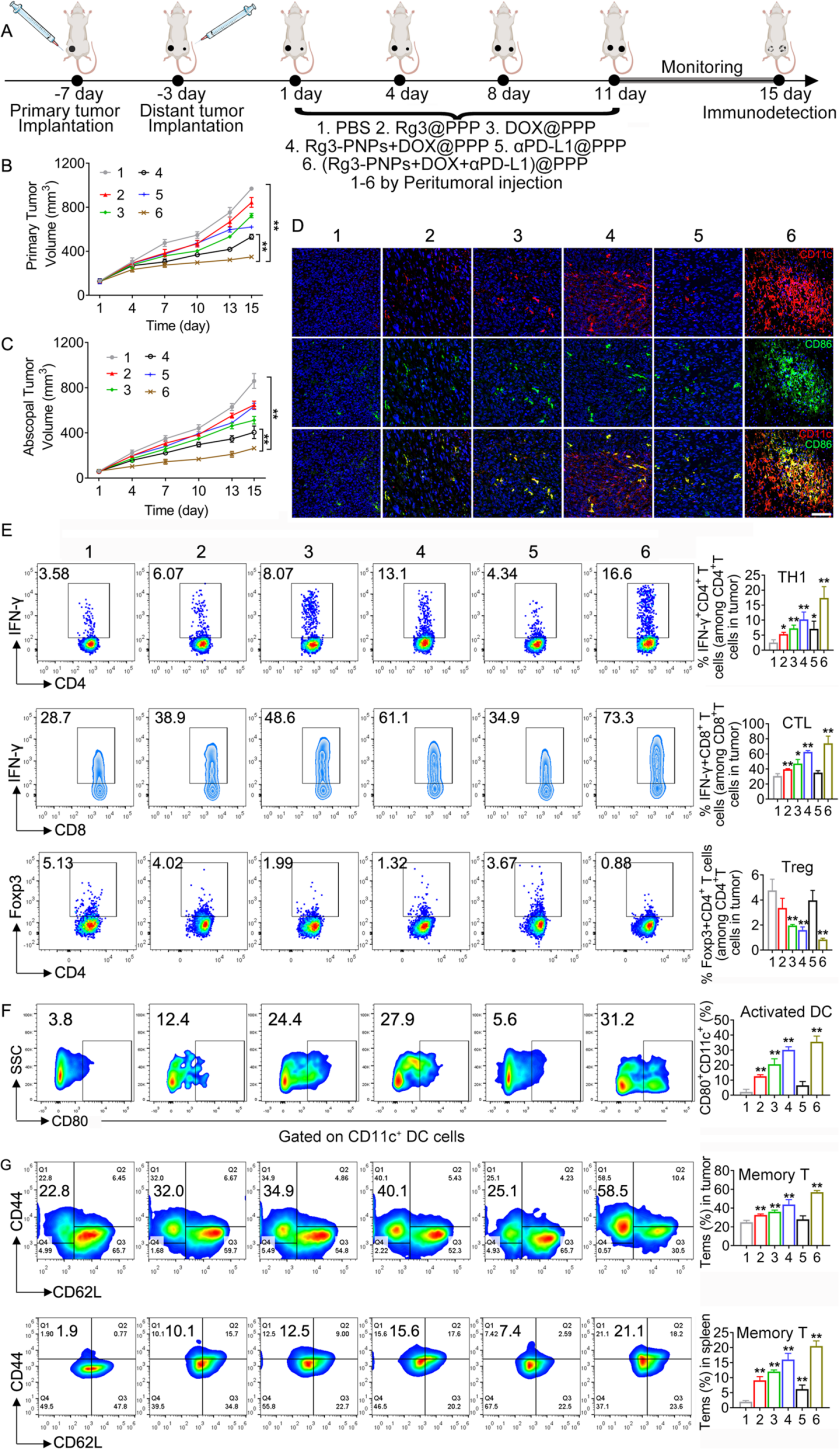
Anti-tumor evaluation on the bilateral 4T1 tumor model to examine the abscopal effect. (**A**) Schedules for the indicated treatment. p.t. means peritumoral. Tumor volume of the primary (**B**) and the distal (**C**) tumors of each group (*n* = 5). (**D**) Immunofluorescence staining of CD11 (red) and CD86 (green) in distal tumors. (**E**) Recruitment of TH1 (CD4^+^IFN-γ^+^ T), CTL (CD8^+^IFN-γ^+^ T) and Treg (CD4^+^Foxp3^+^ T) in distal tumors. (**F**) Populations of CD80^+^ cells gated on CD11c^+^ DC cells in distal tumors. Representative flow cytometry profiles and quantitation of effector memory T cells (CD3^+^CD8^+^CD44^+^CD62L^−^) in the (**G**) distal tumors and in the (**H**) spleen of 4T1-bearing mice. Results were expressed as mean ± SD (*n* = 3). (1) PBS; (2) Rg3@PPP; (3) DOX@PPP; (4) Rg3-PNPs + DOX@PPP; (5) αPD-L1@PPP; (6) (Rg3-PNPs + DOX + αPD-L1)@PPP. ^*^p < 0.05, ^**^p < 0.01 vs. PBS group

We further verified whether the active immune responses were strong enough to constrain an untreated distal tumor. This effect was observed by establishing the bilateral tumors model (Fig. 8A). After peritumoral treatment on primary tumors, the volume of the distal tumors was measured. Strikingly, the growth of distal tumors was considerably slowed down on the mice treated with hydrogels containing Rg3-PNPs + DOX + αPD-L1 (Fig. 8C). As shown in immunofluorescence staining, the combo hydrogel therapy promoted CD86^+^ and CD11c^+^ positive cell numbers in the tumors sections (Fig. 8D). Accordingly, similar trends were observed in CD80^+^DC (activated DC markers) proportion in the tumor by flow cytometry analysis (Fig. 8F). Meanwhile, single αPD-L1 treatment caused no evident DC maturation and activation, explaining why αPD-L1 alone could not prevent tumor growth.

The immunosurveillance cells including TH1 (IFN-γ^+^CD4^+^ T) and CTL (IFN-γ^+^CD8^+^ T) cells surged in both the distal tumors by flow cytometry analysis, while the immunosuppressive Tregs (CD4^+^Foxp3^+^ T) declined after (Rg3-PNPs + DOX + αPD-L1)@PPP treatment (Fig. 8E). Comparatively, both Rg3@PPP and DOX@PPP displayed partial effect on immune cells. Besides, we also evaluated T cell activation in the spleen, and similar trend was shown in spleen. (Rg3-PNPs + DOX + αPD-L1)@PPP group also performed best in regulating T cell activation (supplementary Fig. S8). The outcome of αPD-L1@PPP treatment alone was limited, indicating that systemic immunity was deserted in the immunosuppressive tumor microenvironment of the distal tumors. Hence, insufficient T cell recruitment is another reason for αPD-L1 therapy failure.

Memory T cells retain specific memory of their encountered antigens. We also detected the population of memory T cells (Tems) in the tumor and in the spleen by flow cytometry. The ratios of tumor Tems (CD3^+^CD8^+^CD44^+^CD62L^−^) in (Rg3-PNPs + DOX + αPD-L1)@PPP group were notably boosted to 58.5%, which were 2.6 folds to that of the PBS group (Fig. 8G). Similar trends were observed in the memory T cell proportion in the spleens (Fig. 8H).

## Discussion

Immunotherapy using checkpoint inhibitors has shown substantial benefit in cancer treatment, but tumors that are devoid of T cells or intrinsically lack antigen presentation are less likely to respond to checkpoint inhibitors [56]. A “cold” tumor is not sensitive to PD-L1 antibody largely owing to the low immunogenicity [57]. One approach to transform cold tumor into hot ones is to induce immunogenic cell death (ICD) within the tumor [58]. ICD, a process that dying tumor cells release immunostimulatory signals, can orchestrate an immunogenic environment and foster the anti-PD-1/PD-L1 therapy efficacy [59]. Numerous clinical trials are evaluating checkpoint inhibitors in combination with chemotherapy, in order to complement anti-PD-L1 blockade [60]. As a potent ICD inducer, Dox has been used clinically in combination with immunotherapies.

Rg3 has been reported to induce ICD against cancer cells both in vitro and in vivo [26]. When Rg3 was combined with DOX, the ICD efficacy was significantly improved, as demonstrated by increased HMGB1 release and extracellular ATP levels. Moreover, CS and R6F3-modified nanoparticles (PNPs) further promoted the ICD-inducing effect by Rg3 and DOX (Fig. 2B, C). Therefore, the anti-tumor of Rg3 modified with R_6_F_3_-CS achieved the strongest antitumor efficacy in combination with DOX, with the IC_50_ value of DOX dropping from 3.045 µg/mL to 0.489 µg/mL (Fig. 2A).

Poor drug penetration can be attributed to the high interstitial fluid pressure [39]. To examine whether R_6_F_3_-CS modification could promote the permeability of PPP formulation, multicellular spheroids composed of activated NIH/3T3 and 4T1 cells were constructed with the diameter ranging from 450 to 550 μm. Coumarin-6 (CUR) was utilized to replace Rg3 to perform the tumor spheroid penetration arrays.

CPPs with arginine have been reported to induce trans-membrane transportation of the carrier [61]. R6F3 is a cell-penetrating peptides with 6 arginine and 3 phenylalanine. After conjugated with the CS, the hydrophobicity of phenylalanine facilitates the self-assembly of R6F3-CS. Besides, arginine could provide efficient transport of drugs to enhance the penetration into tumor, and the positive charge of arginine also contribute to achieve the mitochondrial targeting, thus increasing the penetration ability of the PNPs (Fig. 4 A-D). As the vital cellular organelles, mitochondria play an essential role to produce energy, maintain metabolic homeostasis and are regarded as the critical regulators of cell death in apoptosis [41]. It has been reported that mitochondrial damage induced by drugs can evoke ICD [42], attributable to mitochondria dysfunction and subsequent amplification of oxidative stress [43]. Therefore, the precise mitochondrial targeting in this study may be related to the positive charge of CS (Fig. 4E, F). enhanced penetration and mitochondrial targeting further enhance the role of Rg3 in this drug delivery system.

The dying tumor cells caused by ICD serve as an endogenous vaccine and activate T cells and DC; and subsequently recruit immune cells into lymph node or the tumor. Consistent with previous reports [1], 4T1 tumors were initially “immune deserted”: More Tregs aggregate in the tumor site while CD8^+^ T cells are absent in tumor microenvironment as shown in flow cytometry analysis (Fig. 7B). Potent ICD induction by DOX and Rg3 nanoparticles meaningfully reshaped the anticancer immunity in “cold” 4T1 tumors. Enhanced DC activation and T cell infiltration within the 4T1 tumor was observed in mice treated with Rg3-PNPs + DOX@PPP (Fig. 8D and E). In the bilateral tumor model, peritumorally injected hydrogels containing Rg3-PNPs + DOX + αPD-L1 activated the immune system efficiently (Fig. 8G). The primary tumors receiving treatments displayed a great shrinkage in volume and the growth of distal 4T1 tumors was also successfully repressed (Fig. 8C and 8D).

However, currently known ICD inducers all stimulate tumor cells to express more PD-L1 [62]. DOX can kill 4T1 cells effectively and also results in IFN-γ increment, which has also been reported to increase PD-L1 expression on tumor cells and cause immunosuppression via the PD-1/PD-L1 pathway. In order to escape capture and destruction by immune cells, the residual 4T1 cells adaptively hijack checkpoints, such as PD-L1 [54]. Accordingly, PD-L1 expression was up-regulated in mice treated with DOX@PPP. Rg3 has been reported to suppress the PD-1/PD-L1 pathway in TNBC cells [55, 63]. Therefore, PD-L1 expression was notably lower in Rg3-PNPs + DOX@PPP group than that of DOX@PPP group. Moreover, addition of PD-L1 antibody further abolished the negative feedback of PD-L1 up-regulation caused by DOX chemotherapy. As expected, PD-L1 expression decreased sharply in Rg3-PNPs + DOX@PPP + αPD-L1 group, when compared with DOX@PPP group.

## Conclusion

The immunogenic cell death induced by DOX is relatively weak, failing to provoke satisfactory anti-tumor immune response. In order to achieve advancement in tumor penetration, which greatly influenced the synergistic effect of DOX and Rg3, a self-assembly nanoparticle was developed with CS and the amphiphilic cell-penetrating peptides (R_6_F_3_). We first constructed transmembrane peptides-modified chitosan (R_6_F_3_-CS), and then utilized R_6_F_3_-CS to encapsulate Rg3, namely Rg3-PNPs. The in vitro studies suggested that Rg3-PNPs promoted cytotoxicity and immunogenic cell death induced by DOX. Injectable biodegradable hydrogels have shown great potential as local drug carriers with advantages such as few systemic side effects and sustained drug release profiles. Therefore, the thermo-sensitive hydrogel (PLGA-PEG-PLGA, PPP) was applied for co-encapsulation of Rg3-PNPs and DOX. PNPs@PPP formulation displayed mitochondrial targeting and achieved a deep tumor penetration in multicellular spheroids. The local chemotherapy Rg3-PNPs + DOX@PPP augmented the ICD effect in mice, via CRT translocation and HMGB1 release, which further stimulated DC maturation to phagocytize dead tumor cells, and to present antigens. Rg3-PNPs + DOX@PPP, combined with αPD-L1, inhibited bilateral tumor growth, extended mice survival, remodeled immunosuppressive microenvironment and induced a robust immune response, including the increment of immunosurveillance cells TH1 and CTLs, and the decrement of Treg cells. In summary, this thermosensitive hydrogel holds promise as a localized drug-delivery platform for improving the anticancer efficacy of chemoimmunotherapy.

## Supplementary Information

**Additional file 1:** **Supplementary Figure S1. **Schematicprocedure of the synthesis of PEP-CS. **Supplementary Figure S2.** The characterization ofCS, Pep and Pep-CS. (a) The FT-IR of CS, Pep and Pep-CS. The ^1^H NMR spectra of CS (b) and the ^1^H NMR spectra ofPep-CS (c). **Supplementary Figure S3.** The stability of Rg3-NPsand Rg3-PNPs (pH 7.4) at 25°C for up to 7 days. **Supplementary Figure S4.** The stability of blank PPPand Rg3-PNPs+DOX@PPP (pH 7.4) at 25°C for up to 7 days. **Supplementary Figure S5. **4T1cytotoxicity study and CI value treated with different combined groups. **Supplementary Figure S6.** The degradation behaviorof the thermogel and the H&E-stained images of skin tissue aftersubcutaneous injection of thermogel. **Supplementary Figure S7. **Body weight and histopathologicalimages of the major organs, including spleen, lung, liver, heart and kidneyobtained from the 4T1-bearing mice sacrificed at Day 15 (scale bar: 100 μm). **Supplementary Figure S8.** Representative flow cytometry profiles and quantitation of TH1 (IFN-γ^+^CD4^+^T) cell, CTL (IFN-γ^+^CD8^+^T) cell, and Tregs (CD4^+^Foxp3^+^T) cell populations in spleen from tumor-bearing mice. The data show mean ± SDfrom a representative experiment (n=3). *p < 0.05, ** *p* < 0.01 vs PBS. **Supplementary Table S1**. Properties of different nanoparticles. **Supplementary Table S2.**Zeta potential of blank PPP and Rg3-PNPs+DOX@PPP.

## Data Availability

For data requests, please contact the authors.
