# Supplementary material for: Ginsenoside Rg3 nanoparticles with permeation enhancing based chitosan derivatives were encapsulated with doxorubicin by thermosensitive hydrogel and anti-cancer evaluation of peritumoral hydrogel injection combined with PD-L1 antibody
Source: Biomater Res. 2022 Dec 9;26:77. doi: 10.1186/s40824-022-00329-8 (PMC9733157; doi:10.1186/s40824-022-00329-8)
Supplement: Supplementary file 1 — Additional file 1: Supplementary Figure S1. Schematicprocedure of the synthesis of PEP-CS. Supplementary Figure S2. The characterization ofCS, Pep and Pep-CS. (a) The FT-IR of CS, Pep and Pep-CS. The 1H NMR spectra of CS (b) and the 1H NMR spectra ofPep-CS (c). Supplementary Figure S3. The stability of Rg3-NPsand Rg3-PNPs (pH 7.4) at 25°C for up to 7 days. Supplementary Figure S4. The stability of blank PPPand Rg3-PNPs+DOX@PPP (pH 7.4) at 25°C for up to 7 days. Supplementary Figure S5. 4T1cytotoxicity study and CI value treated with different combined groups. Supplementary Figure S6. The degradation behaviorof the thermogel and the H&E-stained images of skin tissue aftersubcutaneous injection of thermogel. Supplementary Figure S7. Body weight and histopathologicalimages of the major organs, including spleen, lung, liver, heart and kidneyobtained from the 4T1-bearing mice sacrificed at Day 15 (scale bar: 100 μm). Supplementary Figure S8. Representative flow cytometry profiles and quantitation of TH1 (IFN-γ+CD4+T) cell, CTL (IFN-γ+CD8+T) cell, and Tregs (CD4+Foxp3+T) cell populations in spleen from tumor-bearing mice. The data show mean ± SDfrom a representative experiment (n=3). *p < 0.05, ** p < 0.01 vs PBS. Supplementary Table S1. Properties of different nanoparticles. Supplementary Table S2.Zeta potential of blank PPP and Rg3-PNPs+DOX@PPP. [file 40824_2022_329_MOESM1_ESM.docx]

**Supplementary Information**

**Ginsenoside Rg3 nanoparticles with permeation enhancing based chitosan derivatives were encapsulated with doxorubicin by thermosensitive hydrogel and anti-cancer evaluation of peritumoral hydrogel injection combined with PD-L1 antibody**

Hao Wu ^c^, Guoli Wei ^a, b, d^, Lixia Luo ^a, b^, Lingchang Li ^a, b^, Yibo Gao ^a, b^, Xiaobin Tan ^a, b^, Sen Wang ^a, b^, Haoxiao Chang ^c, e^, Yuxi Liu ^c^, Yingjie Wei ^a, b^, Jie Song ^a, b,^ *, Zhenhai Zhang ^a, b,^ *, Jiege Huo ^a, b,^*

a *Affiliated Hospital of Integrated Traditional Chinese and Western Medicine, Nanjing University of Chinese Medicine, 210023 Nanjing, China*

b *Jiangsu Province Academy of Traditional Chinese Medicine, 210028 Nanjing, China*

c *School of Material Science and Chemical Engineering, Chuzhou University, Chuzhou 239000*

d *Department of Oncology, Nanjing Lishui District Hospital of Traditional Chinese Medicine, Nanjing, China*

e *Department of Neurology, Beijing Tiantan Hospital, Capital Medical University, Beijing, China.*

**Supplementary Methods**

**In vivo Hydrogel Degradation.** The thermosensitive hydrogel (PLGA-PEG-PLGA, PPP) (20%, 200 μL) was injected into the right backs of BALB/c mice. The mice were photographed and sacrificed at different time intervals. The remaining hydrogel in each mouse was firstly photographed. Skins were examined by H&E staining.

**Supplementary Results**


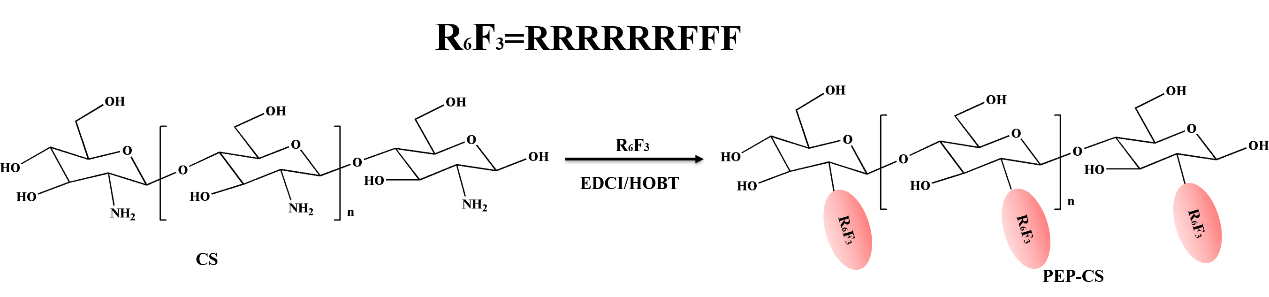


**Supplementary Figure S1.** Schematic procedure of the synthesis of PEP-CS.


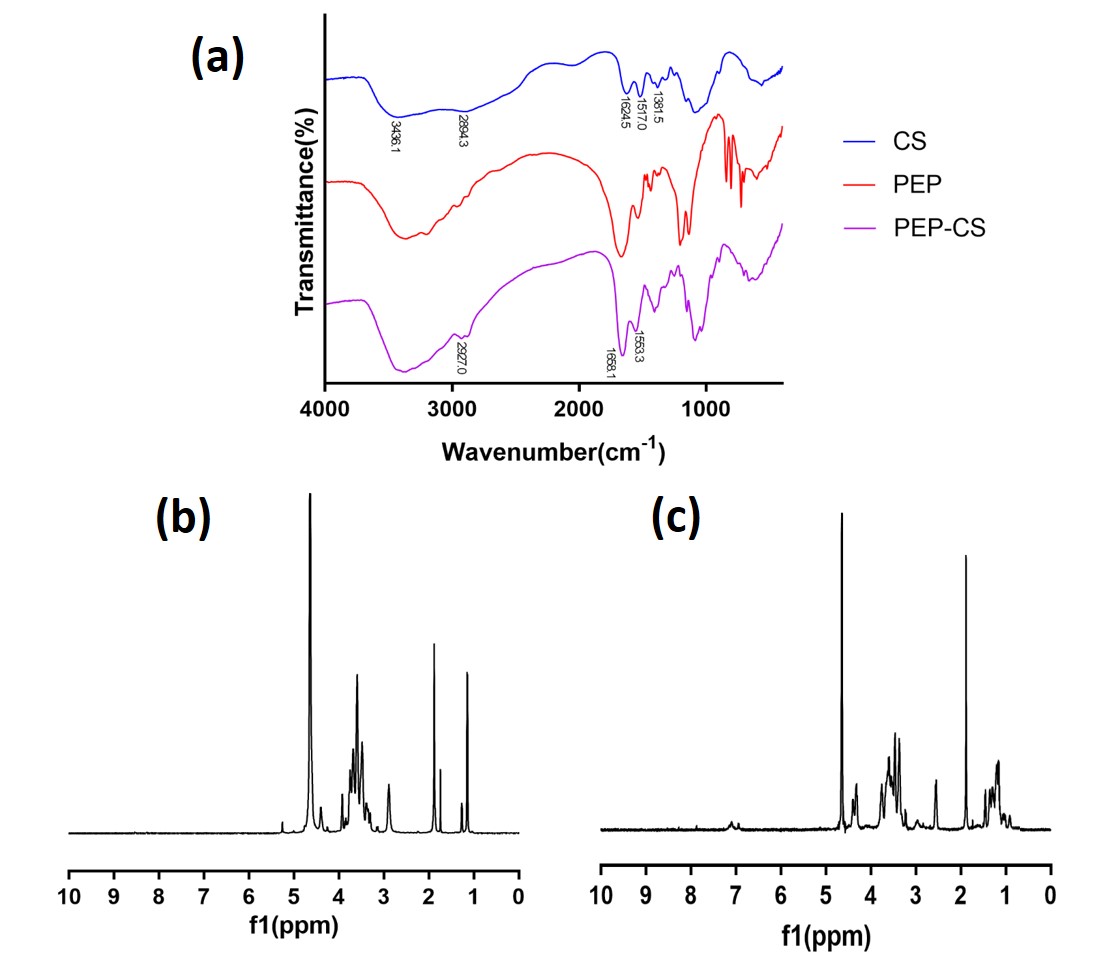


**Supplementary Figure S2.** The characterization of CS, Pep and Pep-CS. (a) The FT-IR of CS, Pep and Pep-CS. The ^1^H NMR spectra of CS (b) and the ^1^H NMR spectra of Pep-CS (c).


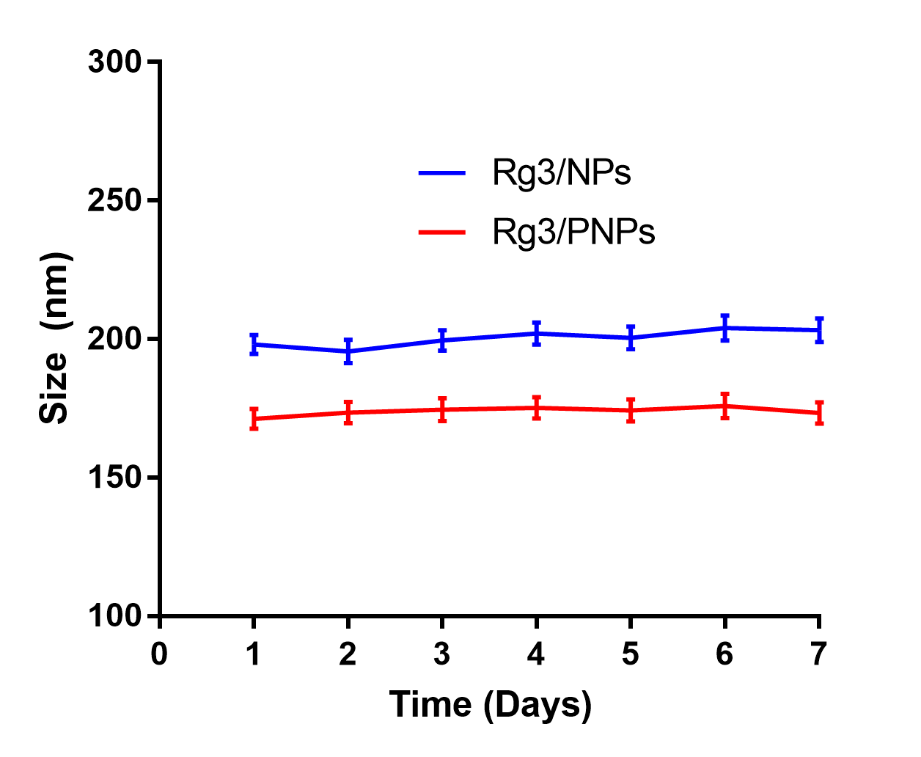


**Supplementary Figure S3.** The stability of Rg3-NPs and Rg3-PNPs (pH 7.4) at 25°C for up to 7 days.


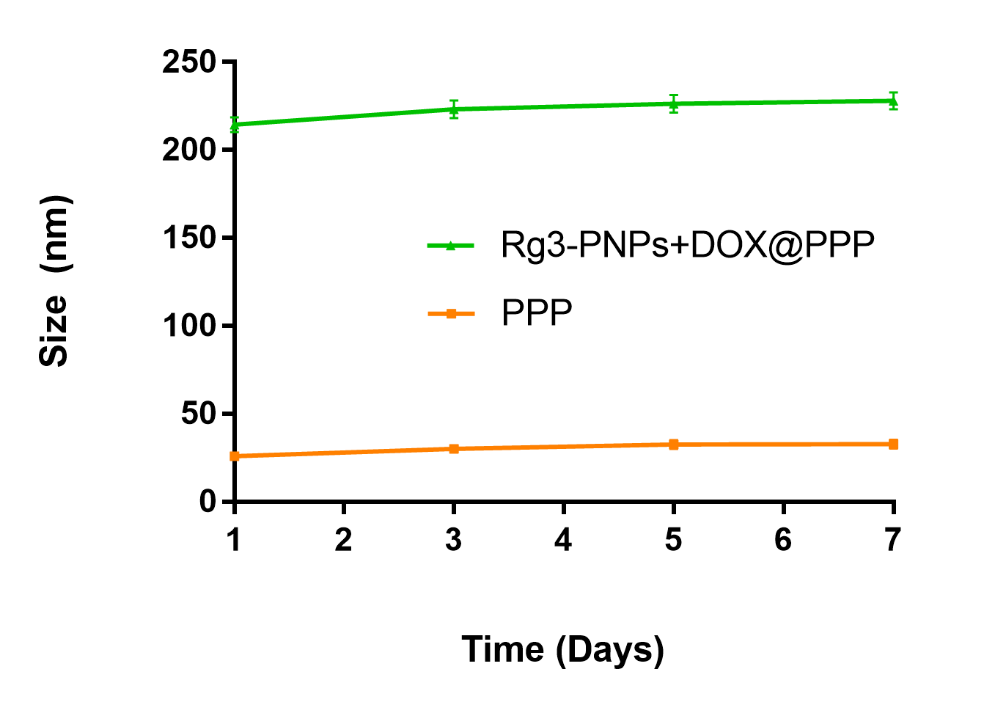


**Supplementary Figure S4.** The stability of blank PPP and Rg3-PNPs+DOX@PPP (pH 7.4) at 25°C for up to 7 days.


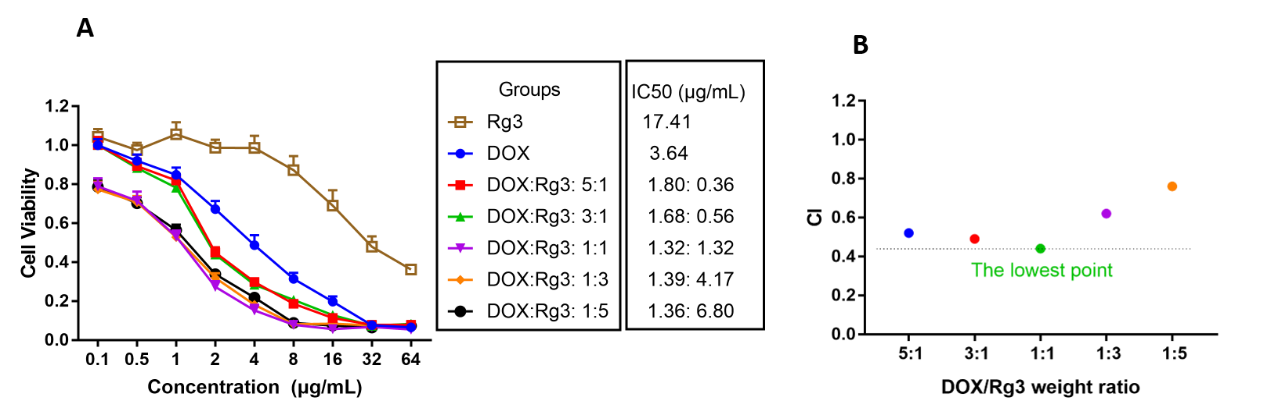


**Supplementary Figure S5** 4T1 cytotoxicity study and CI value treated with different combined groups.


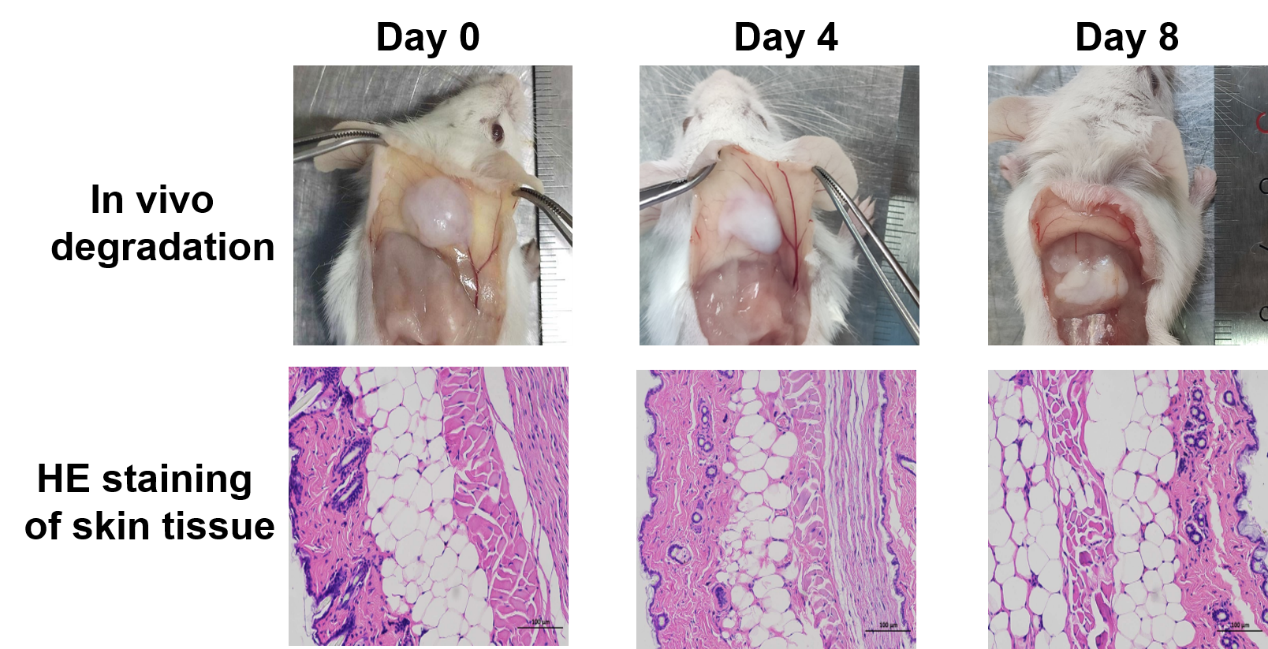


**Supplementary Figure S6.** The degradation behavior of the thermogel and the H&E-stained images of skin tissue after subcutaneous injection of thermogel.


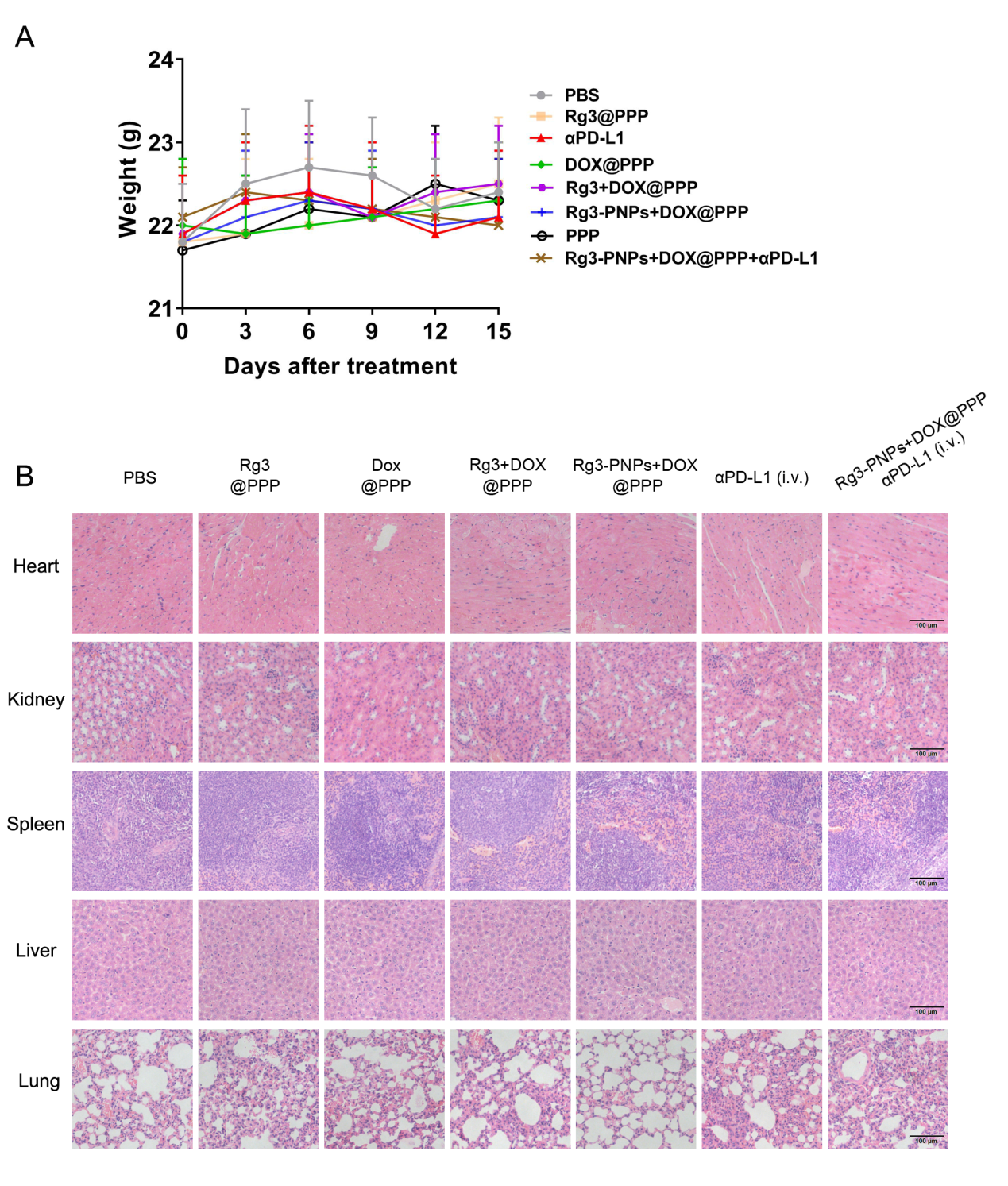


**Supplementary Figure S7.** Body weight and histopathological images of the major organs, including spleen, lung, liver, heart and kidney obtained from the 4T1-bearing mice sacrificed at Day 15 (scale bar: 100 μm).


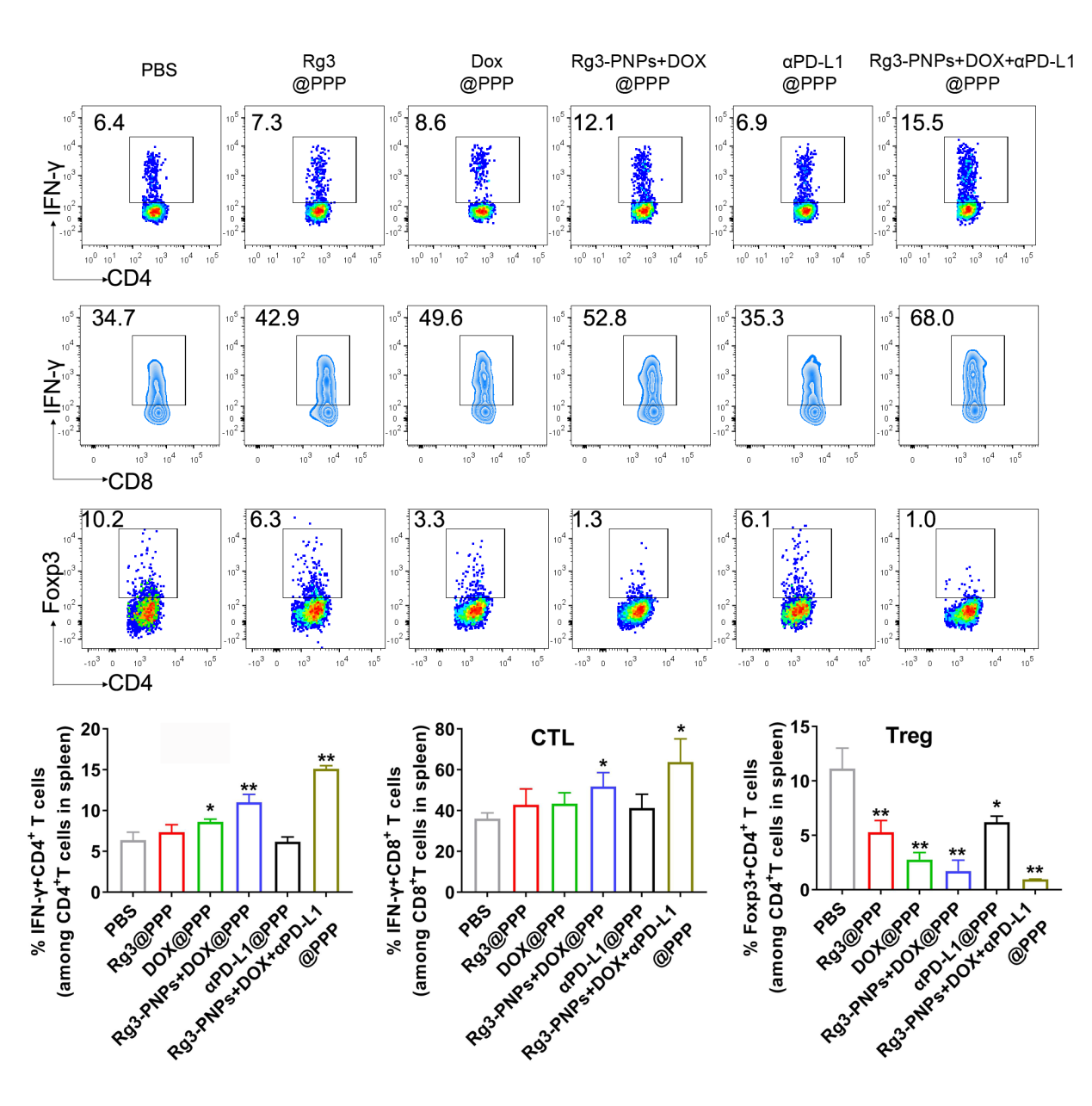


**Supplementary Figure S8.** Representative flow cytometry profiles and quantitation of TH1 (IFN-γ^+^CD4^+^T) cell, CTL (IFN-γ^+^CD8^+^T) cell, and Tregs (CD4^+^Foxp3^+^ T) cell populations in spleen from tumor-bearing mice. The data show mean ± SD from a representative experiment (n=3). *p < 0.05, ** p < 0.01 vs PBS.

**Supplementary Table S1**. Properties of different nanoparticles.

| Sample | DL (%) | EE (%) |
| --- | --- | --- |
| Rg3-NPs | 8.7±1.2 | 50.3±2.3 |
| Rg3-PNPs | 9.2±1.7 | 53.6±1.8 |

The data are presented as means ± SD (*n* = 3).

**Supplementary Table S2.** Zeta potential of blank PPP and Rg3-PNPs+DOX@PPP

| Time (day) | PPP | Rg3-PNPs+DOX@PPP |
| --- | --- | --- |
| 1 | -0.51±0.08 | 10.7±0.76 |
| 3 | -0.54±0.07 | 9.8±0.73 |
| 5 | -0.57±0.07 | 11.3±0.79 |
| 7 | -0.63±0.06 | 11.7±0.81 |
